# Flavour by design: food-grade lactic acid bacteria improve the volatile aroma spectrum of oat milk, sunflower seed milk, pea milk, and faba milk towards improved flavour and sensory perception

**DOI:** 10.1186/s12934-023-02147-6

**Published:** 2023-07-21

**Authors:** Muzi Tangyu, Michel Fritz, Jan Patrick Tan, Lijuan Ye, Christoph J. Bolten, Biljana Bogicevic, Christoph Wittmann

**Affiliations:** 1 Institute of Systems Biotechnology, Saarland University, Saarbrücken, Germany; 2 Nestlé Research Center, Lausanne, Switzerland; 3 Nestlé Product Technology Center Food, Singen, Germany

**Keywords:** Lactic acid bacteria, Fermentation, Flavour, Off-flavour, Legumes, Plant-based milk, Oat milk, Sunflower seed milk, Pea milk, Faba milk, GC‒MS, Volatiles, Odour activity

## Abstract

**Background:**

The global market of plant-based milk alternatives is continually growing. Flavour and taste have a key impact on consumers’ selection of plant-based beverages. Unfortunately, natural plant milks have only limited acceptance. Their typically bean-like and grassy notes are perceived as “off-flavours” by consumers, while preferred fruity, buttery, and cheesy notes are missing. In this regard, fermentation of plant milk by lactic acid bacteria (LAB) appears to be an appealing option to improve aroma and taste.

**Results:**

In this work, we systematically studied LAB fermentation of plant milk. For this purpose, we evaluated 15 food-approved LAB strains to ferment 4 different plant milks: oat milk (representing cereal-based milk), sunflower seed milk (representing seed-based milk), and pea and faba milk (representing legume-based milk). Using GC‒MS analysis, flavour changes during anaerobic fermentations were studied in detail. These revealed species-related and plant milk-related differences and highlighted several well-performing strains delivered a range of beneficial flavour changes. A developed data model estimated the impact of individual flavour compounds using sensory scores and predicted the overall flavour note of fermented and nonfermented samples. Selected sensory perception tests validated the model and allowed us to bridge compositional changes in the flavour profile with consumer response.

**Conclusion:**

Specific strain-milk combinations provided quite different flavour notes. This opens further developments towards plant-based products with improved flavour, including cheesy and buttery notes, as well as other innovative products in the future. *S. thermophilus* emerged as a well-performing strain that delivered preferred buttery notes in all tested plant milks. The GC‒MS-based data model was found to be helpful in predicting sensory perception, and its further refinement and application promise enhanced potential to upgrade fermentation approaches to flavour-by-design strategies.

**Supplementary Information:**

The online version contains supplementary material available at 10.1186/s12934-023-02147-6.

## Background

The global market of plant-based milk alternatives (referred to here as plant milks) is continually growing. Valued at US$ 20.5 billion in 2020, this market is expected to expand at a calculated annual growth rate (CAGR) of 12.5% over the next years [1]. Notably, 40% of consumers are willing to reduce the use of animal-based protein because of environmental concerns [2]. In addition to these environmental considerations, increasing plant milk consumption is driven by increased attentiveness to animal welfare, health-related issues, such as lactose intolerance and milk allergies, and lifestyle changes [3]. To fully meet consumer expectations in terms of product quality, plant milks are intended to resemble animal milk and provide pleasant flavour and taste [4]. However, plant-based milks typically exhibit a bean-like flavour and bitter taste, refused by many consumers. These notes are perceived as undesired “off-flavour” [5–7]. The undesirable flavour is largely due to the presence of certain aldehydes, alcohols, and ketones in the plant materials [8], including hexanal, *n*-hexanol, and ethyl vinyl ketone [9].

Different physico-chemical treatments have used microwaves [10], cold plasma [11], soaking [12], and blanching [13] to remove these off-notes from plant milks. In addition, natural fermentation by food-grade microbes has emerged as an appealing option to improve the flavour of plant-based milks and, at the same time, increase nutritional value, stability, and microbial safety [3, 6, 14, 15]. Lactic acid bacteria (LAB) are regarded as particularly promising. These microbes have a long tradition in fermenting dairy products and have proven valuable in plant-based milk fermentation. For example, species of *Streptococcus, Lactococcus, Lacticaseibacillus*, *Lactiplantibacillus*, *Lactobacillus, Limosilactobacillus,* and *Leuconostoc* were shown to affect the flavour of cereal-based milk [16, 17], chickpea milk [18], sunflower seed milk [7], soy milk [19, 20], mung bean milk [21], cowpea milk [22], pea-based materials [23, 24], and fruity and vegetable juice [25]. Therefore, fermentation decreased bean-like and grassy note volatiles and completely removed hexanal [20], while selected strains additionally formed buttery [26, 27] and cheesy aroma compounds [28, 29].

Following this promising potential, we evaluated a range of LAB to ferment four different emerging plant milks: oat milk (representing cereal-based milk), sunflower seed milk (representing seed-based milk), and pea and faba milk (representing legume-based milk). The four plant milks were selected based on industrial impact, considering availability in big volume, sustainability, cost, and nutrition (protein quality). As example, oat milk is becoming the second-most consumed plant milk due its high protein content, dairy-like taste, and environmental sustainability [30, 31]. Likewise, faba [32] and pea milk [33] are emerging as sustainable quality plant protein sources. Sunflower seed milk, obtained from cheap residuals of sunflower oil manufacturing, does not provide protein of the same high quality but appears particularly attractive in terms of sustainability and economic potential [34]. For clean label products, the raw materials were applied as a simple suspension in water without further supplementation.

Using solid-phase microextraction and GC‒MS analysis with automated data deconvolution, flavour changes during fermentation were studied at the molecular level, revealing species-related and plant milk-related differences and, notably, highlighting several well-performing strains that delivered beneficial flavour changes. For several of these well-performers, we predicted perceivable sensory impressions of the fermented plant milks based on odour activities of the contained volatiles using a data-driven model. The predictions matched the outcome of sensory evaluations by a panel of test persons, which allowed us to bridge compositional changes with consumer response. In this regard, our work provides a comprehensive understanding of LAB-related flavour alteration in various plant-based nutrient environments. Moreover, specific strain-milk combinations were identified that open further developments towards cheesy products, buttery products, and other innovative products in the future.

## Results

### Characterization of unfermented plant-based milks on the level of nutrient composition and flavour-contributing organic volatiles

The two major aims of this work were to (i) evaluate and improve the undesired flavour of plant milks using microbial fermentation and (ii) discover beneficial microbes that perform well, independent of the plant milk type and origin. On the raw material side, plain plant milks were used, i.e., aqueous suspensions of the plant material without additional supplements [35]. The gross nutrient composition of the four selected materials differed substantially (Table 1). Protein represented the largest fraction (2.0–4.3%), followed by carbohydrates (0.3–3.5%) and fat (0.1–2.7%). Oat milk exhibited the highest content of the major nutrients [36, 37]. In contrast, pea milk and faba milk contained relatively low levels of protein (approximately 2.0%) and carbohydrates (0.3% and 0.4%, respectively). Sunflower seed milk exhibited intermediate levels of protein (3.6%) and carbohydrates (0.7%), while its fat level was negligible because defatted sunflower seed flour had been used for preparation [7].

**Table 1:** Nutrient composition of oat milk, sunflower seed milk, pea milk, and faba milk

	Protein (%)	Carbohydrate (%)	Fat (%)
Oat milk	4.30	3.50	2.70
Sunflower seed milk	3.60	0.70	0.05
Pea milk	2.00	0.29	0.14
Faba milk	2.20	0.41	0.12

**Fig. 1:**
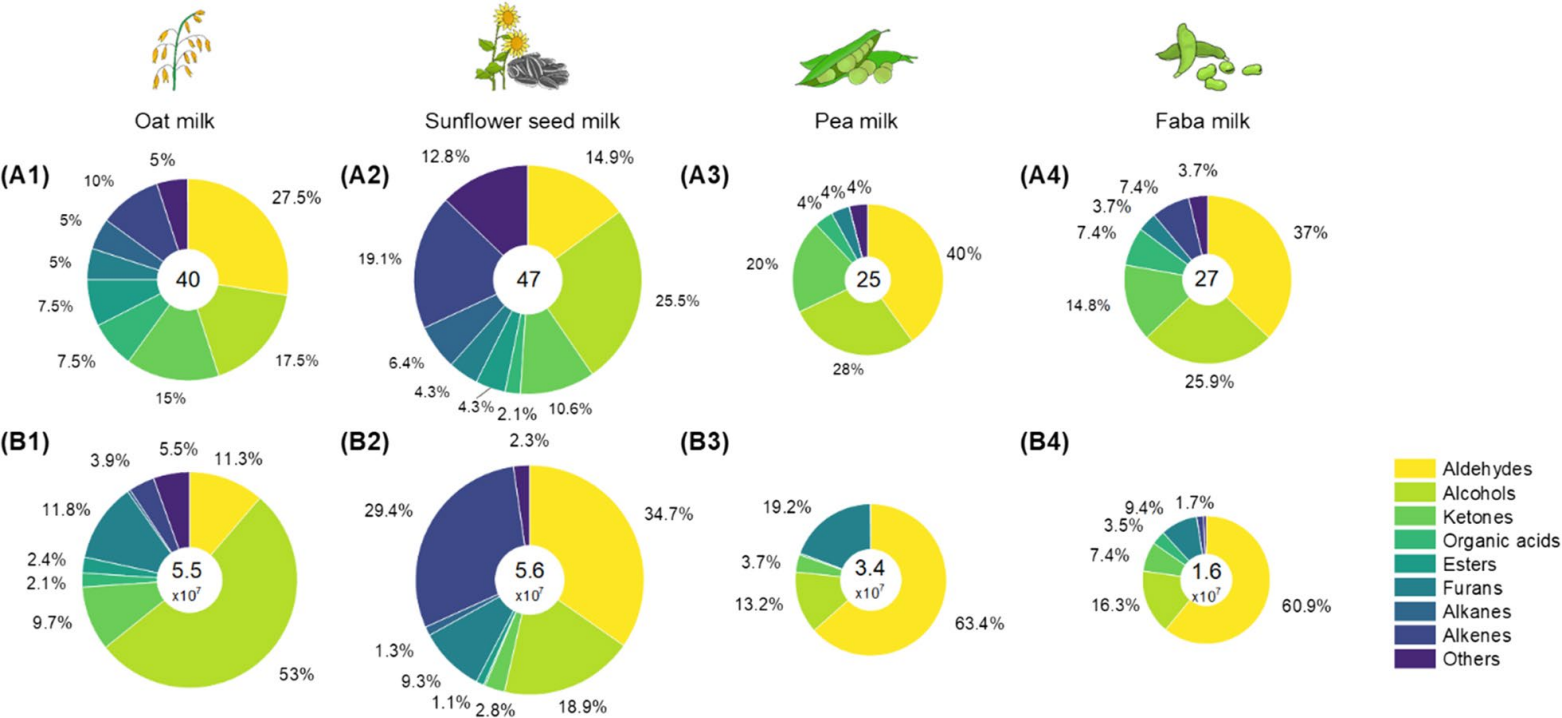
Volatile analysis of nonfermented oat milk (1), sunflower seed milk (2), pea milk (3), and faba milk (4) using GC‒MS. The data comprise the number of known detected volatile compounds (**A**) and the relative peak area (**B**) associated with specific chemical groups. The relative diameter of each pie graph represents the corresponding number (**A**) and the total peak area of all volatiles detected (**B**). The values are additionally shown in the middle of each chart. n = 3

Next, the volatile flavour profile of the raw materials was evaluated (Fig. 1, Table 2, Additional file 1). For this purpose, we used SPME via a DVB/CAR/PDMS fibre plus GC‒MS (Additional file 2: Table S1). Generally, the measurement showed high reproducibility (standard deviation < 20%). Overall, 71 volatiles were detected and identified, the most in sunflower seed milk and the least in legume-based milks, well matching previous observations and confirming the suitability of the developed analytics [36, 38–43]. Oat milk contained volatiles from different chemical groups, whereby alcohols (18% of all detected volatiles, 53% of the total peak area), aldehydes (28% of all volatiles, 11% of the total peak area), and ketones (15% of all volatiles, 10% of the total peak area) were dominant. At the level of single compounds, 1-hexanol, 2-pentylfuran, and dimethyl ether were found to be most abundant, all attributed to undesired bean-like flavour (Table 2). These three molecules accounted for 42%, 12%, and 5% of the total peak area, respectively. In contrast, sunflower seed milk was found to be rich in alkenes, especially terpinene-based volatiles such as α-pinene and β-terpinene, which accounted for 23% of the total peak area. Furthermore, aldehydes and alcohols were predominant, including 1-hexanal (23% of the total peak area) and 1-hexanol (10% of the total peak area). The two legume-based milks from pea and faba showed fewer volatiles. Aldehydes (37–44% of all volatiles) were detected in both plant milks, followed by alcohols (26–28%), whereas unlike the other plant milks, no esters or alkanes were present. All plant milks contained substantial levels of hexanal, the primary aldehyde attributed to bean-like flavour [8].

**Table 2:** Identified volatile compounds in unfermented and fermented plant-based milks, aroma attributes, odour groups, and the odour threshold in air

Nr	Compound name^a^	RI, cal (min)	Aroma description	Odour group^b^	Odour threshold (ppbv)	Identification
Aldehydes						
1	3-Methyl-butanal	648	Malty, fatty, cocoa, fruity	1	11	MS, RI
2	2-Methyl-butanal	654	Malty, cocoa	1	11	MS, RI
3	Hexanal	799	Green, grassy, nutty, fat, oxidized oil	6	0.28	MS, STD, RI
4	2,4-Heptadienal^3^	1010	Fatty, creamy, green	3	8	MS, RI
5	2-Heptenal	957	Green, fatty	6	19	MS, RI
6	Heptanal	904	Green, citrus, fatty, floral, rancid	6	0.18	MS, STD, RI
7	Benzaldehyde	959	Sweet, fruity, almond	1	20	MS, STD, RI
8	2-Octenal	1058	Fatty	3	0.53	MS, RI
9	Phenylacetaldehyde	1043	Floral, sweet, honey	2	4	MS, STD, RI
10	4-Ethyl-benzaldehyde	1161	Fruity	1	13	MS, RI
11	2-Nonenal	1158	Green, musty, fatty,	6	0.02	MS, RI
12	Nonanal	1103	Citrus, floral, fatty, green, smoky	1	0.34	MS, STD, RI
13	2,4-Decadienal^4^	1312	Grass, fatty, melon, aldehyde	6	0.07	MS, RI
14	Decanal	1202	Fatty, floral	3	0.10	MS, RI
Alcohols						
1	[S, S]-2,3-Butanediol^1,2^	780	Creamy, buttery	3	49	MS, RI
2	3-Methyl-2-buten-1-ol ^2^	773	Fruity	1	173	MS, RI
3	3-Methyl-1-butanol ^2,4^	733	Fruity, banana, whiskey, floral, fermented	1	68	MS, RI
4	2-Methyl-1-butanol ^1,4^	734	Ethereal, floral	1	68	MS, RI
5	1-Pentanol	765	Fermented, sweet, fruity, balsamic, alcoholic	1	43	MS, STD, RI
6	1-Hexanol	869	Fruity, lemon	1	6	MS, STD, RI
7	2-Heptanol^1,3,4^	900	Citrus, fruity, herbal	1	41	MS, RI
8	1-Heptanol	970	Light green, mushroom, rancid	6	3	MS, STD, RI
9	Benzyl alcohol^2,3^	1033	Floral	2	1 × 10^4^	MS, RI
10	2-Methyl-3-hexanol^1^	859	Unknown	9		MS, RI
11	Phenylethyl alcohol^2,4^	1112	Floral, rose, honey	2	0.02	MS, STD, RI
12	1-Octen-3-ol	979	Earthy, mushroom	7	0.52	MS, RI
13	2-Octen-1-ol^1,2,3,4^	1067	Cucumber	2	40	MS, RI
14	3-Octanol^4^	996	Earthy	7	27	MS, RI
15	2-Ethyl-1-hexanol^4^	1028	Citrus, floral	1	130	MS, RI
16	1-Octanol	1070	Waxy, aldehyde, fruity, floral	3	2.7	MS, RI
17	1-Nonanol^2,4^	1171	Citrus, rose	1	0.9	MS, RI
18	Eugenol^4^	1368	Pleasant spicy, clove-like	2	6	MS, STD, RI
19	Cherry-propanol	1183	Fruity	1		MS, RI
20	Trans-pinocarveol	1138	Woody, balsamic	5		MS, RI
21	*cis*-Verbenol	1166	Balsamic, pine	5		MS, RI
22	(-)-Myrtenol	1195	Woody, minty, camphoraceous	5	0.32	MS, RI
23	Myrtenol	1203	Woody, herbal, floral	5	0.32	MS, RI
24	Linalool	1100	Floral, fruity	2	4	MS, RI
25	Terpinen-4-ol	1176	Balsamic, woody, green, fatty, fruity, floral, spicy	5	150	MS, RI
26	3-Ethyl-4-nonanol^2,3^	1094	Unknown	9		MS, RI
Ketones						
1	Methyl-vinyl-ketone	734	Pungent, sweet	8		MS
2	2,3-Butanedione^2,3,4^	440	Buttery, sweet, creamy	3	0.3	MS, RI
3	3-Hydroxybutan-2-one^2,4^	706	Buttery, creamy, sweet, toasted	3	0.3	MS, RI
4	2,3-Pentanedione^3^	694	Buttery, sweet	3	12	MS, RI
5	2-Hexanone^1, 2^	800	Fruity	1	24	MS, RI
6	2,3-Heptanedione^1^	836	Buttery	3		MS, RI
7	3,6-Heptanedione^2^	1062	Buttery	3		MS, RI
8	2-Heptanone	891	Fruity, floral, sweet, cheesy	1	0.76	MS, RI
9	1-(2-Furanyl)-1-propanone^2^	1031	Fruity	1		MS, RI
10	3,5-Octadien-2-one	1092	Fruity, fatty, green, earthy	1	5	MS, RI
11	6-Methyl-5-hepten-2-one^3^	987	Citrus, musty, green	1	1	MS, RI
12	5-Methyl-3-hepten-2-one	1039	Unknown	9		MS, RI
13	3-Octanone^4^	987	Earthy [1], mushroom-like [3], fresh [3], herbal [3], ripe banana [3]	7	21	MS, RI
14	Isoacetovanillone	1158	Unknown	9		MS, RI
15	2-Nonanone^4^	1091	Sweet, fruity, floral, green, hot milk, soap	1	5	MS, RI
16	5,6-Dehydrocamphor	1095	Unknown	9		MS, RI
17	Pinocarvone	1162	Camphoraceous, fresh	5		MS, RI
18	D-Verbenone	1208	Camphoraceous, minty, spicy	5		MS, RI
Organic acids						
1	Acetic acid^1,2,3,4^	401	Acidic, sour, vinegar	4	6	MS, STD, RI
2	3-Methyl-butanoic acid^1,3^	843	Cheesy	4	0.08	MS, RI
3	2-Methyl-butanoic acid^2,3^	853	Sour, cheesy, fermented	4	0.04	MS, RI
4	Pentanoic acid^1^	882	Cheesy, acidic, unpleasant	4	8.90	MS, RI
5	Hexanoic acid^2,3^	982	Cheesy, fatty, sour, sharp, rancid	4	0.60	MS, RI
6	Octanoic acid^2,3^	1167	Cheesy, fatty, sweaty	4	27	MS, RI
7	Nonanoic acid	1269	Waxy, earthy	3	1.9	MS, RI
Esters						
1	Ethylacetate	916	Sweet, fruity, mild	1	870	MS, RI
2	Ethyllactate^1^	791	Fruity, buttery	1	5 × 10^4^	MS, RI
3	Ethyl 2-methylbutyrate	850	Fruity	1	0.01	MS, RI
4	Hexyl acetate	1014	Fruity, sweet	1	1.8	MS, RI
5	Verbenylacetate	1144	Unknown	9		MS, RI
6	Bornyl acetate^2^	1201	Balsamic, camphoraceous	5	75	MS
7	Epoxy-alpha-terpenylacetate	1130	Unknown	9		MS, RI
Furans						
1	2-Ethyl-furan^3^	699	Chemical, sweet, coffee-like	8	1.3 × 10^6^	MS, RI
2	2-Ethyl-5-methylfuran^2^	773	Grassy	8		MS, RI
3	2-Acetyl-5-methylfuran	855	Nutty	5		MS, RI
4	2-n-Butyl furan^1^	892	Spicy, fruity, wine-like	8	1 × 10^5^	MS, RI
5	2-(1-Pentenyl)-furan^3^	1001	Roasted	5		MS, RI
6	2-Pentyl-furan	991	Beany, green, grassy, nutty, fatty	6	3.4	MS, RI
7	2-n-Heptylfuran	1190	Green, fatty	6		MS, RI
Alkanes						
1	Undecane	1098	Unknown	9	620	MS, STD, RI
2	Dodecane	1196	Unknown	9	110	MS, STD, RI
3	Tridecane	1295	Unknown	9		MS, STD, RI
Alkenes						
1	α -Pinene	933	Pine, woody, herbal, fresh, fruity	5	18	MS, STD, RI
2	Camphene	950	Woody, herbal, camphoraceous	5	130	MS, RI
3	β-Terpinene	975	Terpenic, fatty	5	130	MS, RI
4	3-Carene	1009	Citrus, sweet	1	140	MS, RI
5	p-Cymene	1024	Fruity, fresh, citrus, terpenic, floral, fragrant	1	57	MS, RI
6	D-Limonene	1028	Citrus, fresh, sweet	1	38	MS, STD, RI
7	γ-Terpinene	1058	Terpenic, citrus, herbal	5	5 × 10^4^	MS, RI
8	α-Thujene^2^	1121	Woody, herbal, green	5		MS, RI
9	Alloocimene^1^	1128	Floral, sweet, nut	2	1.8 × 10^4^	MS, RI
10	β-Thujene	953	Unknown			MS, RI
11	2,4-Dimethyl-1-decene	1083	Unknown	9		MS, RI
12	β-Gurjunene	1434	Unknown	9		MS, RI
Others						
1	Dimethyl ether^2,3,4^	478	Ethereal	1	5 × 10^5^	MS, RI
2	4-Ethenyl-1,2-dimethyl-benzene	1088	Unknown	9		MS, RI
3	4-Methyl-2-propylphenol^1^	1314	Unknown	9		MS, RI
4	α-Limonene-di-epoxide	1031	Citrus	1		MS, RI
5	Unknown^3^	735				
6	Unknown	989				
7	Unknown^4^	1028				
8	Unknown	1039				
9	Unknown	1079				
10	Unknown	1085				
11	Unknown	1332				
12	Unknown	1341				

An extended version that additionally includes the literature for the aroma descriptors and the odor thresholds is available in the Additional file 2: Table S7

^a^The superscripted number represents newly formed volatiles during fermentation of oat milk (1), sunflower seed milk (2), pea milk (3), and faba milk (4)

^b^Odour groups: (1) fruity, citrus, sweet, malty, ethereal; (2) floral (3) buttery, fatty, waxy, creamy; (4) cheesy, sour; (5) nutty, woody, minty, toasted, turpentine, balsamic, camphoraceous; (6) green, grassy, bean-like, herbal; (7) earthy, mushroom; (8) pungent, spicy, sharp, phenolic; and (9) unknown odour

**Fig. 2:**
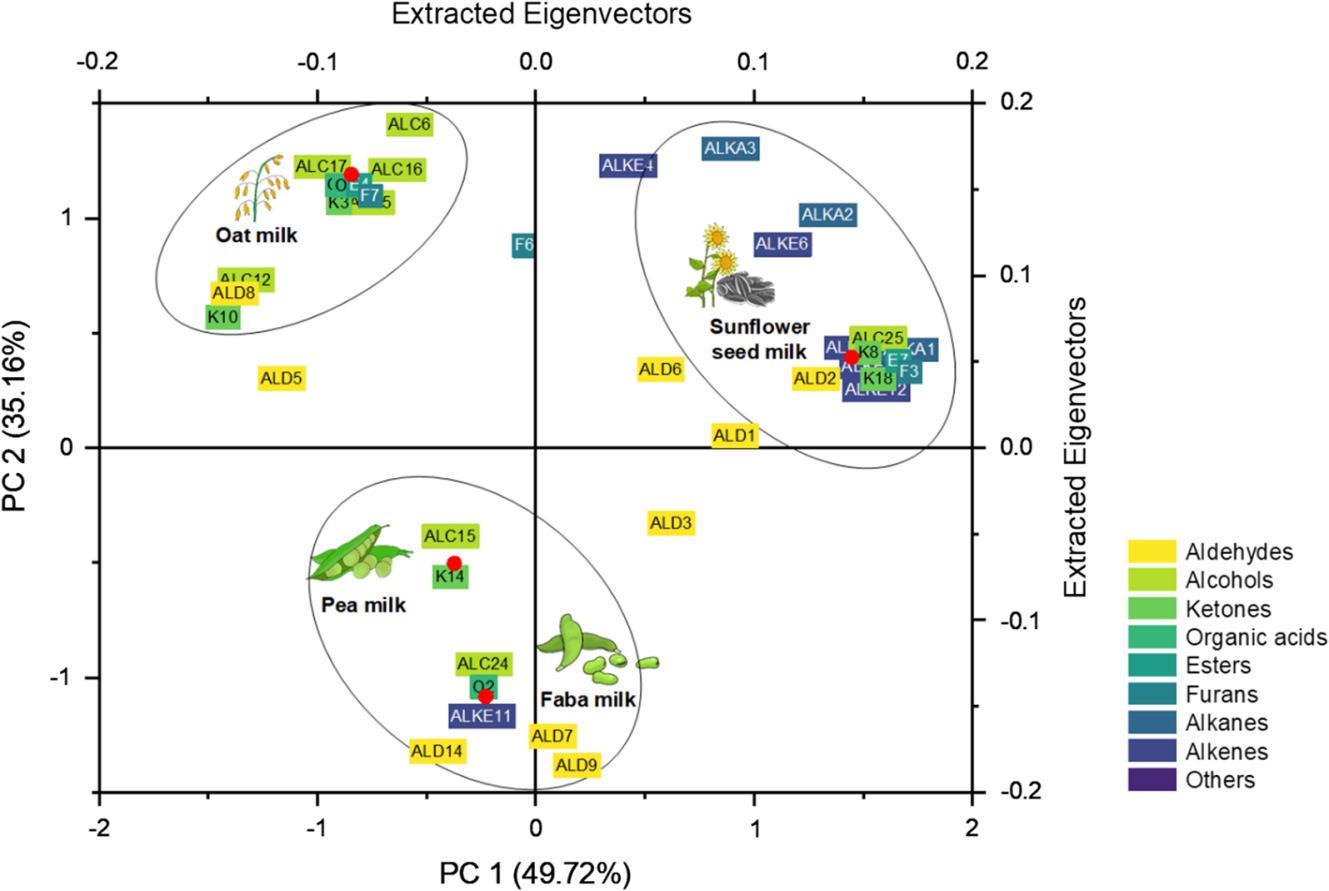
Principal component analysis of the volatiles detected by GC‒MS in unfermented oat milk, sunflower seed milk, pea milk, and faba milk. The data are shown as biplots, including a few signature loading points. *ALC* alcohols, *ALD* aldehydes, *ALKA* alkanes, *ALKE* alkenes, *E* esters, *F* furans, *K* ketones, *O* organic acids. The given number of each compound corresponds to Table 2 (n = 3)

For increased interpretability, the flavour data were subjected to PCA. The differences in volatile abundance led to a clear separation of the data in the obtained PCA plots (Fig. 2)**.** The unfermented plant milks clustered into three groups: (i) cereal-based oat milk, (ii) seed-based sunflower seed milk, and (iii) the two legume-based milks (pea and faba). Alkenes such as α-pinene, β-terpinene, and camphene appeared as signature volatiles of sunflower seed milk, largely contributing to its unique profile. Oat milk and legume-based milks were well separated by differences in the levels of alcohols, ketones, and esters (1-heptanol, 1-hexanol, 2,3-butanedione, ethyl-2-methyl-butanoic acid, and others), as well as aldehydes (phenylacetaldehyde, decanal, benzaldehyde, 1-hexanal) and terpinene-based alcohols (e.g., linalool). Oat milk was rich in alcohols, ketones, and esters, while pea milk and faba milk were characterized by a high aldehyde content.

### Screening of a collection of LAB strains for their plant milk fermentation capacity

On the microbial side, 15 food-grade LAB were selected from two major LAB families, namely, *Streptococcaceae* and *Lactobacillaceae* (Additional file 2: Table S2). The isolates covered eight different genera: *Streptococcus* (4), *Lactococcus* (2), *Lacticaseibacillus* (3), *Lactiplantibacillus* (1), *Lactilactobacillus* (1), *Lactobacillus* (2), *Limosilactobacillus* (1), and *Leuconostoc* (1), which have previously proven value in affecting the flavour of plant-based food in general [26, 36, 44–46]. From a metabolic viewpoint, the selected microbes comprised mostly homolactic LAB, e.g., *Streptococcus* and *Lactococcus*, and a heterofermentative LAB, *Leuconostoc*.

It could be expected that the flavour metabolism of the isolates would require their growth, linked to the fact that the degradation, formation, and interconversion of aroma molecules requires energy, redox power, and building blocks, which are all provided in actively growing cells [47]. The extent of growth strongly varied with strain and plant milk (Table 3). *L*. *helveticus* NCC 1276 could not grow in any of the four milks within the test range of 24 h. All other strains grew in more than one plant milk, whereby the increase in living cells ranged from 0.05 to 2.43 log cfu mL^−1^ (Table 3), reflecting a 1.1-fold to 269.2-fold increase in living cell number. Most *Lactobacillaceae* grew well, while *Streptococcaceae* showed weaker growth. *L. mesenteroides* NCC 2832 revealed strong growth in all plant milks, whereas the other strains showed preferences for specific types of milk. As an example, *L. fermentum* NCC 660, *S. thermophilus* NCC 2019, and *S. thermophilus* NCC 2059 grew well in oat and sunflower seed milk but did not grow, or did so only weakly, in legume-based milk. In contrast, *L. rhamnosus* NCC 2891 and NCC 4007*, L. lactis* NCC 2180 and NCC 2242, and *L. paracasei* NCC 2511 grew well only in oat milk and pea milk. Four strains of *S. thermophilus*, *L. johnsonii* NCC 533, and *L. sakei* NCC 1692 preferred sunflower seed milk, while *L. plantarum* NCC 2988 was the second-best grower in sunflower seed, pea, and faba milk among all strains but grew poorly in oat milk. Obviously, the nutrient composition of the milk had a strong impact on growth. Generally, oat milk enabled better growth than legume-based milks, likely due to the higher protein level [8]. Furthermore, *Lactobacillaceae* grew better than *Streptococcaceae,* eventually linked to their evolutionary adaptation to plant materials [48]. Notably, a few isolates stood out, including *L. mesenteroides* NCC 2832, the only strain with a heterofermentative metabolism [49], exhibiting the best growth. Overall, a range of promising combinations of strains and plant milks could be identified.

**Table 3:**
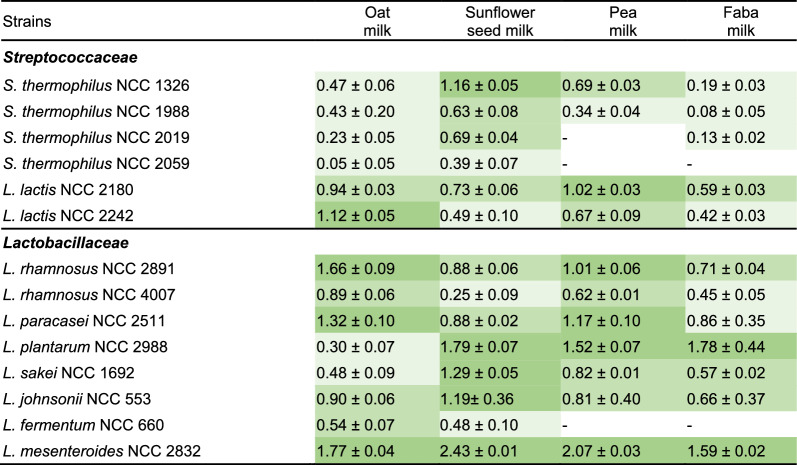
Growth of LAB strains in oat milk, sunflower seed milk, pea milk, and faba milk, expressed as increase in log (cfu mL^−1^) (n=3)

Light green, slight growth (0 < log increase < 0.5); green (0.5 < log increase < 1); dark green (log increase > 1); -, no growth. The data are mean values and standard errors from three biological replicates

### Impact of LAB-based fermentation on the profile of flavour-associated volatiles

**Fig. 3:**
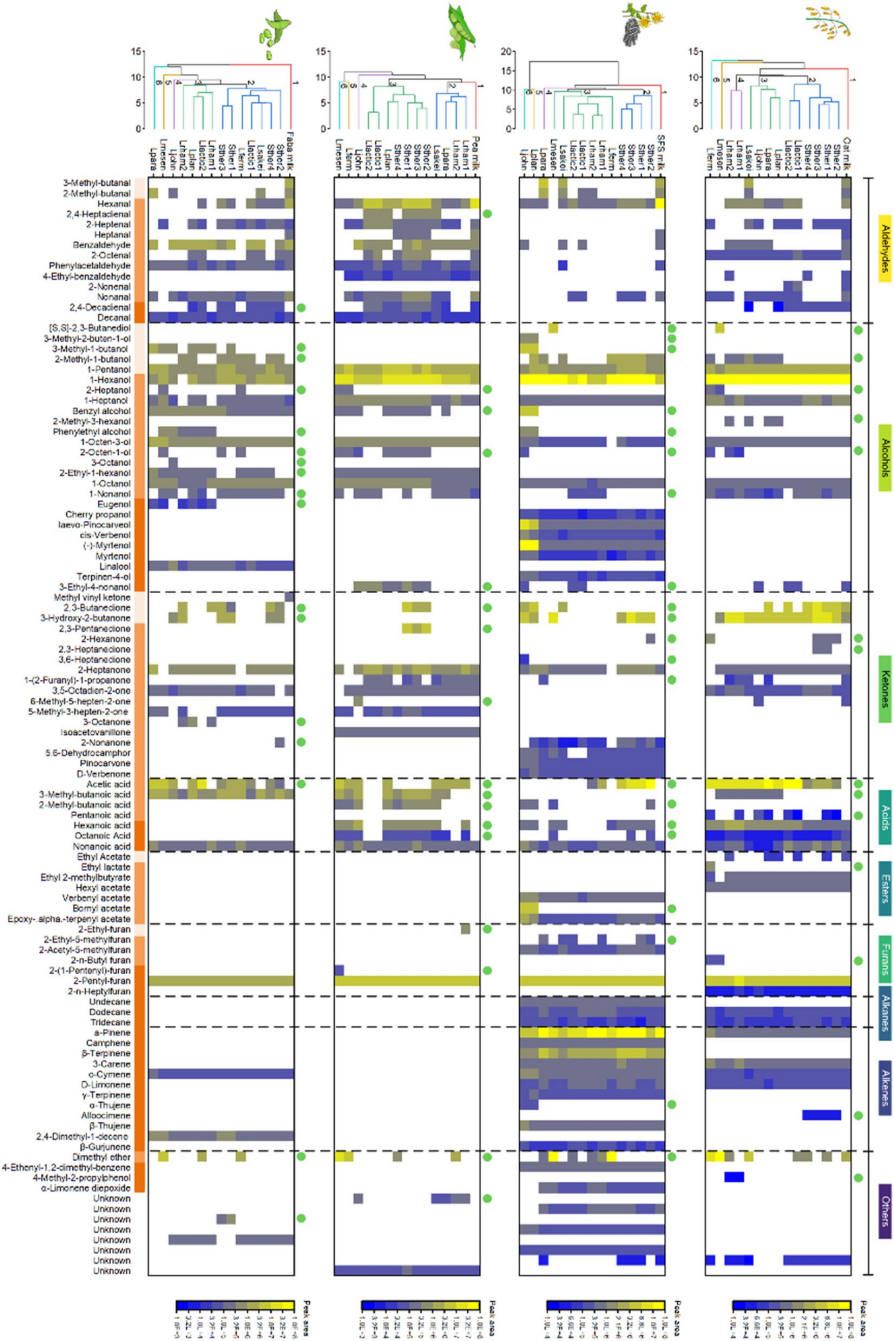
Changes in individual flavour compounds during the fermentation of faba milk, pea milk, sunflower seed, and milk oat milk (from left to right). The abundance of each flavour compound is shown by colour boxes that indicate the observed average peak area: yellow, high abundance; blue, low abundance; and white, not detectable. The flavour compounds are grouped into aldehydes, alcohols, ketones, acids, esters, furans, alkanes, alkenes, and others with the separation of dotted lines. Within each chemical group, the compounds are sorted from small to large: light orange, < C_5_; orange, C_6_—C_9_; strong orange, > C_10_. Newly formed flavour compounds, detected after fermentation, are labelled with green dots. For each milk, the columns are sorted based on hierarchical clustering analysis. For each plant milk, the 14 fermented samples and the nonfermented sample clustered into 6 groups with distances of 6 (oat milk), 9.6 (sunflower seed milk), 9.0 (pea milk), and 8.4 (faba milk) (n = 3)

In the next step, the fermented plant milks were analysed for changes in the spectrum of aroma compound volatiles. For this purpose, volatiles were extracted after 24 h of fermentation using SPME, analysed by GC‒MS, and compared to nonfermented milks. Fermentation strongly affected the volatile spectrum (Fig. 3, Table 2, Additional file 1). Most aldehydes and some ketones present in the nonfermented plant milks were found to be strongly decreased (e. g. 1-heptanal, 1-hexanal, and 1-nonanal). In turn, the corresponding alcohols (1-heptanol, 1-hexanol, 1-nonanol) and carboxylic acids (heptanoic acid, hexanoic acid, nonanoic acid) increased, revealing that the LAB catalysed oxidative and reductive aldehyde conversions. In addition, esters, ketones, and ethers increased notably, matching at least the trend observed in other LAB-based plant fermentations [18, 36, 45, 50].

The two fermented legume-based milks were enriched in aldehydes, acids, and alcohols but rather poor in other aroma compounds. In contrast, fermented oat and sunflower seed milk were lower in aldehydes and richer in ketones, alcohols, acids, and alkenes.

In addition, 27 different volatiles emerged during fermentation: 14 novel compounds were detected in fermented oat milk, 17 in fermented pea and faba milk, and 21 in fermented sunflower seed milk (Fig. 3). Fermented sunflower seed milk exhibited the most diverse aroma profile. Most of the newly formed molecules were small. As an example, newly arising alcohols and ketones comprised four to nine carbon atoms. As an exception, eugenol and 3-ethyl-4-nonanol, bornyl acetate, α-thujene and alloocimene, formed by selected microbes, exhibited 10–13 carbon atoms. As aldehydes were presumed to contribute to undesired bean-like and grassy off-flavour, alcohols were often found to be related to favoured sweet and fruity notes (Table 2), and the observed changes appeared as a first sign for a beneficially affected flavour profile. Hierarchical clustering grouped unfermented plant milk far from fermented plant milk, underlying the impact of fermentation on the volatile spectrum. Many of the observed changes were strain specific, leading to considerable differences in the volatile profiles. Taxonomically related isolates seemed to change the aroma similarly, as found, for example, for *S. thermophilus* and *L. lactis*. As an exception, faba milk resulted in larger differences even for closely related strains, which might be related to the different capabilities of the strains to grow on this raw material.

Notably, the studied strains exhibited quite different reducing and oxidizing activities. Many strains showed a remarkable capacity for reduction. *S. thermophilus* isolates formed alcohols (e.g., 1-heptanol, 1-hexanol, 1-octanol, 2-methyl-1-butanol, 1-pentanol). Specific ketones, such as 2,3-butanedione, 3-hydroxy-2-butanone, 2-hexanone, 2,3-pentanedione, 2-heptanone, 5-methyl-3-hepten-2-one, and 2-nonanone were also formed. Similarly, *L. fermentum* formed high amounts of several alcohols, including 2-methyl-1-butanol, 1-heptanol, 1-hexanol, 2-heptanol, 1-heptanol, 2-octen-1-ol, and 1-octanol. In contrast, *L. mesenteroides* formed a greater level of oxidized products such as carboxylic acids (nonanoic acid, hexanoic acid, acetic acid). Some strains exhibited mixed behaviour and catalysed reductions as well as oxidations. As an example, oat milk fermented by *L. rhamnosus* contained specific alcohols (2-octen-1-ol, 1-octanol, 2-methyl-1-butanol), ketones (3-hydroxy-2-butanone, 2-heptanone), and organic acids (acetic acid, 3-methyl-butanoic acid, 2-methyl-butanoic acid, hexanoic acid, pentanoic acid). For some strains, the catalysed chemistry differed with the plant material. In sunflower seed milk and faba milk, *L. johnsonii* NCC553 prominently formed alcohols. In pea milk, the strain produced more organic acids, offering diverse potential for applications in different plant milks. This was an important discovery because choosing strains for fermentation of plant-based materials is a challenging exercise, as the different plant-based matrices can be very different and findings in one are not necessarily transferable to another.

### Statistical analysis of the volatile data highlights strain-specific flavour phenotypes

**Fig. 4:**
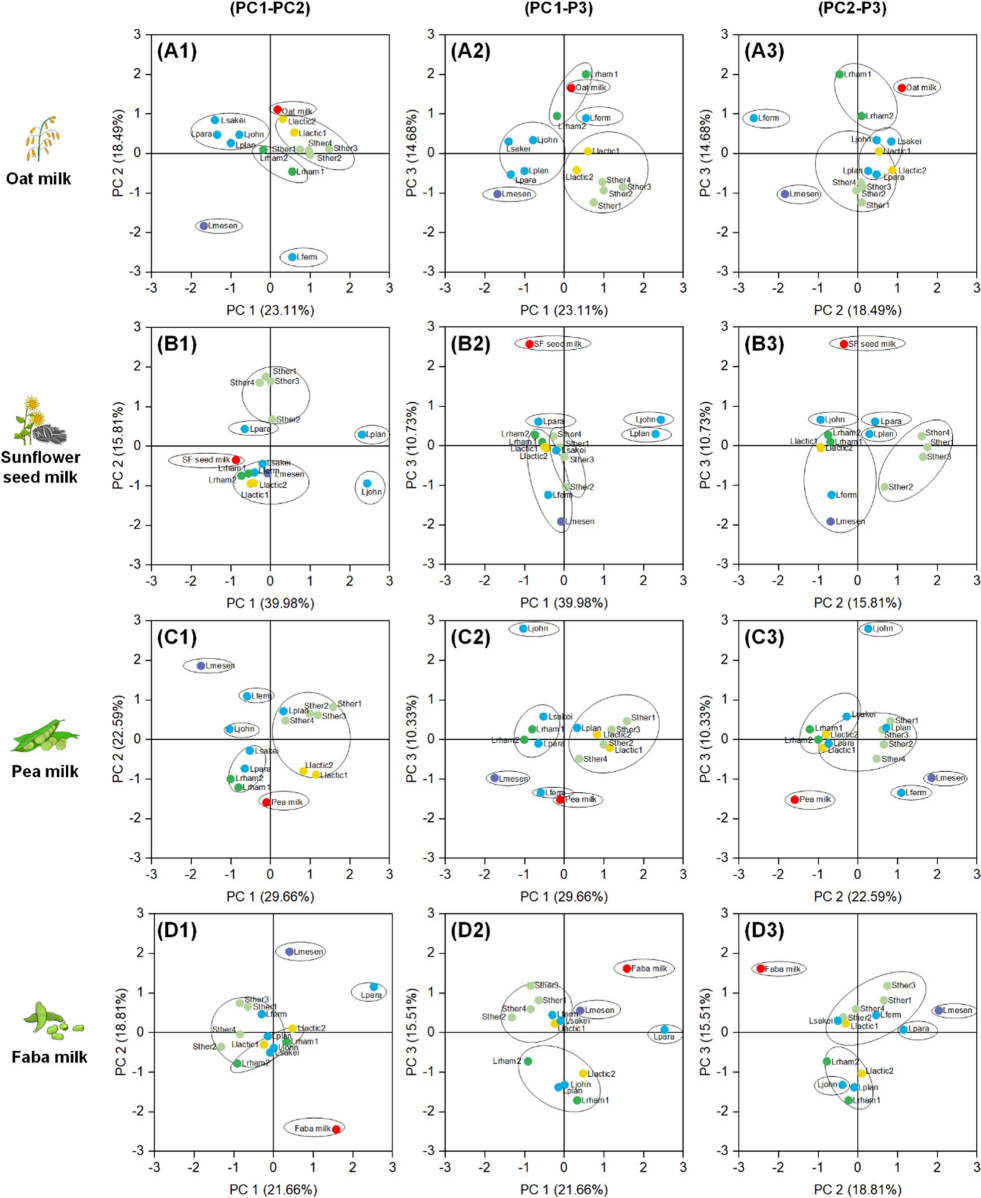
Impact of plant milk fermentation on the volatile spectrum assessed by PCA. The data comprise the results for oat milk (raw 1), sunflower seed milk (raw 2), pea milk (raw 3), and faba milk (raw 4) detected by HS-SPME-GC‒MS. **A**, Score plot of principal components 1 and 2; **B**, Score plot of principal components 1 and 3; **C**, Score plot of principal components 2 and 3. The colour was given to unfermented plant milk and different species: red, unfermented plant milk; light green, *S. thermophilus*; dark green, *L. rhamnosus*; yellow, *L. lactis*, purple, *L. mesenteroides*; blue, others (n = 3)

A more systematic insight into the fermentations was obtained by PCA based on the GC‒MS and the growth data (Additional file 1, Additional file 2: Table S4, S5, S6, S7). The latter allowed us to consider growth effects on flavour changes, a trend that seemed important, based on the hierarchical clustering results (see above). Altogether, 60 unfermented and fermented plant milk samples were analysed. It turned out that the first (PC1), the second (PC2), and the third principal component (PC3) together explained up to 67% of the total variance, so their inspection allowed us to extract important features in the dataset (Fig. 4, Additional file 2: Table S4, S5, S6, S7).

In all cases, unfermented plant milk clustered separately from fermented plant milk, independent of the strain used. The highest distance was observed for sunflower seed, pea, and faba milk (Fig. 4B–D), e.g., along the axes of PC2 and PC3. Unfermented plant milk coclustered with aldehydes, followed by certain organic acids, alcohols, and ketones, meaning that its unique position was due to a high abundance of these compounds. In contrast, the fermented plant milks were mainly characterized by the presence of esters, alkenes, and ethers. In addition, sunflower seed milk clustered separately along the axis of PC3 mainly due to differences in the abundance of aldehydes, alcohols, and alkenes.

At the strain level, different isolates from the same species revealed a similar activity to affect the spectrum of volatiles, at least in some of the principal components. Typically, they clustered together (Fig. 4). For example, the four *S. thermophilus* isolates grouped together in all plant milk samples. In contrast, the two strains of *L. rhamnosus* clustered together in all plant milks except faba milk. Likewise, both *L. lactis* strains clustered together in oat, sunflower seed, and pea milk but were separated in faba milk. Interestingly, the aroma effects caused by *L. lactis* were highly like those of *S. thermophilus*. In contrast, rather pronounced plant milk-based differences were observed for strains *L. fermentum, L. johnsonii, L. paracasei, and L. plantarum*. These strains exhibited a specific response to each nutrient environment, indicating a more individual flavour metabolism and offering more flexibility regarding the desired profile. *L. sakei*, although taxonomically more distant, seems to not provide a significantly different type of flavour. The strain always clustered with other strains so that it appeared replaceable to some extent. Notably, *L. mesenteroides* behaved quite differently. It was located far away from all other microbes, indicating that this microbe created a unique flavour profile among all isolates.

### Estimation of flavour changes from changes in the compositional aroma spectrum during fermentation

**Fig. 5:**
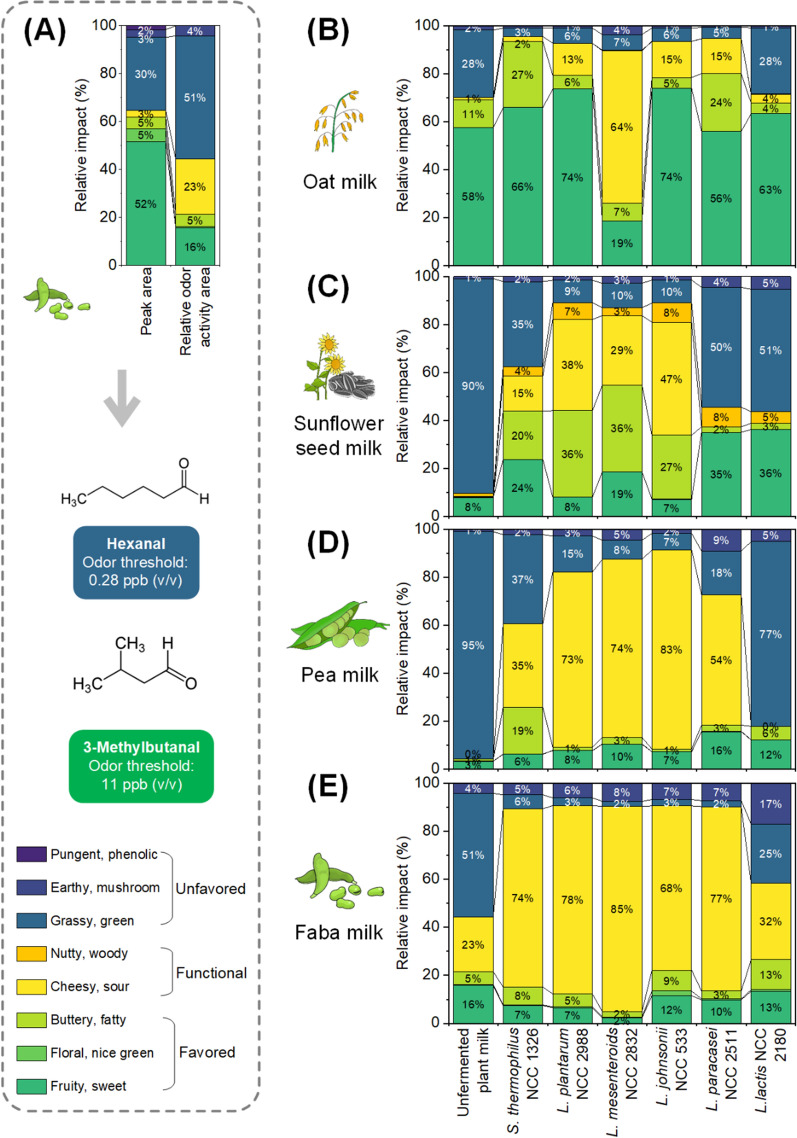
Prediction of perceivable flavour from nonfermented and fermented plant milks based on GC‒MS volatile analysis. Illustration of the concept of inferring relative odour activity from relative abundance using unfermented faba milk as an example (**A**). Prediction of the flavour profile and the overall sensory profile for oat milk (**B**), sunflower seed milk (**C**), pea milk (**D**), and faba milk (**E**). Prior to estimation, the detected volatiles were classified into eight odour groups: (i) Fruity, citrus, sweet, melty, ethereal (designated fruity, sweet); (ii) floral (floral); (iii) buttery, fatty, waxy, creamy (designated buttery, fatty); (iv) cheesy, sour (designated cheesy, sour); (v) nutty, woody, minty, toasted, turpentine, balsamic, camphoraceous (designated nutty, woody); (vi) green, grassy, bean-like, herbal (designated grassy, green); (vii) earthy, mushroom (designated earthy, mushroom); and (viii) pungent, spicy, sharp, phenolic earthy, mushroom pungent, phenolic). The colours highlight desired flavours (green), functional flavours (yellow), and off-flavours (blue) (n = 3)

As shown, fermentation strongly affected the flavour profile of the plant milk. While certain signature molecules could be identified and strains could be classified in terms of similarity and uniqueness, the observed changes were complex. We were, however, interested in translating the compositional changes in the volatile spectra into changes in perceivable flavour. We approached this question by weighing the compositional volatile data based on relative volatile abundance and odour threshold. The approach mimicked the concept of odour activity, which is based on ratios between concentration and odour threshold and is frequently used to infer the impact of single compounds on overall flavour [51]. A test set of six representative strains that yielded quite different flavour profiles, based on PCA and HCA analysis, was used (Fig. 5).

In contrast to the odour activity concept, our approach evaluated the flavour impact of single compounds within a given mixture based on relative peak areas (offering the benefit that these are far more easily accessible than absolute concentrations). In the first step, relative peak areas for each compound were estimated as a fraction of the total peak area of all detected compounds. Then, the relative peak areas were subjected to weighing, considering the individual odour threshold, i.e., the minimum concentration of a substance at which a majority of test subjects can detect and identify its characteristic odour. Therefore, the least perceivable volatile within the sample was used as a reference, and the other (more perceivable) volatiles were normalized to this reference based on the threshold ratio. This yielded the relative odour activity for each compound within the mixture, i.e., its impact on flavour.

In total, 69 out of 98 detected volatiles with known flavour attributes, for which a threshold value was available, could be included in the evaluation. Then, we estimated the overall flavour note of a sample. For this purpose, we summed all relative odour activity values that belonged to a certain flavour group (Fig. 5A). Doing so for different flavour groups finally aimed to provide a rough estimate of the resulting overall flavour that we designated “estimated flavour” here. Sunflower seed milk, pea milk, and faba milk are well known to have a strong grassy and bean-like flavour [7, 39, 52], which is most pronounced for pea milk [53]. It was therefore nice to see that our approach exactly predicted this attribute. The three unfermented plant milks were dominated by a grassy and bean-like note: 51% for faba milk, 90% for sunflower seed milk, and 95% for pea milk. The predicted undesired flavour note was consistent with previous studies [54–56]. As shown, fruity notes made only a minor contribution to the flavour of these plant milks. In contrast, unfermented oat milk was classified to be more fruity-sweet and buttery-fatty (69% of total odour impact), and these beneficial attributes well matched previous consumer perception studies of taste [57].

Using our concept, several interesting findings could be extracted from the fermentation data. For pea, faba, and sunflower seed milk, fermentation with most strains resulted in drastic alteration of the estimated flavour, whereas for oat milk, only one strain (*L. mesenteroides*) caused an apparently strong change (Fig. 5). Within the flavour fingerprints, we recognized notable trends among (i) favoured plant milk flavours such as sweet, floral, and buttery (shown green); (ii) functional flavours that add complexity and uniqueness to the food [58], such as minty, cheesy, and nutty (shown yellow); (iii) and generally unfavoured flavours such as grassy and bean-like (shown in blue). Most strains could substantially increase favoured flavours (fruity, sweet, floral, buttery, and fatty). Strikingly, *S. thermophilus* NCC 1326 was able to increase the buttery note in all four plant milks. This feature was regarded as very attractive, given that this note is generally preferred in fermented plant milk products. The highest increase in favoured flavours was observed for fermented oat and sunflower seed milk, but fermented pea and faba milk also contained elevated amounts of these flavours. Nutty, woody, and minty notes increased in fermented sunflower seed milk, driven by the availability of corresponding flavour precursors and the capacity of the strains used. Cheesy and sour odours were increased in fermented pea milk and faba milk, demonstrating the huge range of different flavours that was achieved.

Most beneficially, all strains were able to remove unfavoured bean-like and grassy volatiles, in some combinations even drastically. As an example, *L. johnsonii* reduced the faction of these notes from 95 to 7% in pea milk. Among all strains, *L. plantarum*, *L. mesenteroides*, and *L. johnsonii* were found to be most efficient in decreasing this off-flavour, providing a valuable trait [53]. In contrast, *L. lactis* was weaker in lowering the bean-like and grassy notes, likely linked to its rather poor capacity to metabolize aldehydes [26].

### Bridging GC‒MS-predicted odour implication and multisensory flavour perception

As shown, fruity/sweet, buttery/fatty, cheesy/sour, and grassy/green notes were the four most dominant flavour groups, as estimated from the GC‒MS data for unfermented and fermented plant milk, and thus assumed to largely contribute to sensory perception. These apparently most relevant changes were finally evaluated by sensory tests of an untrained panel. The setup was as follows. First, oat milk, fermented by *S. thermophilus* NCC 1326, *L. plantarum* NCC 2988, *L. mesenteroides* NCC 2832, *L. paracasei* NCC 2511, *L. lactic* NCC 2180, and *L. johnsonii* NCC 533, as described before, was analysed for its most pronounced increase in fruity/sweet and buttery/fatty notes. Second, pea milk fermented by the same strains was evaluated for its presumable increase in cheesy/sour aroma and the decrease in grassy/green notes, as predicted by the GC‒MS data. Generally, differences in favoured fruity/sweet and buttery/fatty notes between unfermented and fermented oat milk were well noticed by the panel.

## Discussion

### LAB fermentation removes undesired volatile aldehydes and increases the level of fruity terpenoid-based flavour compounds

Plant-based milk alternatives are rising in popularity and sales, given their ecological, ethical, and environmental benefits. The rise could be even stronger if these products were to taste better. Admittedly, their undesired bean-like flavour acts a main hurdle to global consumer adoption [59]. In this regard, our study considered four emerging plant milks, which each offer attractive properties. Oat milk consumption reduces blood cholesterol due to its high fibre content, providing an important health benefit [60], whereas sunflower seed milk, produced from press cakes, inexpensive residuals from sunflower oil manufacturing, offers attractive sustainability and economic potential [34]. Pea- and faba-based milk displays a high-quality protein source due to the elevated abundance of essential amino acids [61]. However, all four plant-based milks suffer from undesired sensory attributes [3].

To improve flavour, we explored 15 different LAB strains from different families and genera for their potential to modify the spectrum of flavour-contributing volatiles during fermentation of the selected plant-based milks. As shown, fermentation resulted in substantial changes in the profile of flavour-contributing nutrients.

In contrast to previous work, we did not supplement additional nutrients (such as sugars or animal milk) [14, 37, 62, 63] or used mixed cultures [14, 37, 64] to support the growth of the LAB. Fourteen out of the selected 15 strains could grow as mono-cultures on more than one plant milk without any supplement, providing promising potential towards clean label products. It should, however, be noted that the efficiency of growth differed for the different strains and eventually impacted flavour formation. In this regard, the weaker formation of favoured flavours in the legume-based milks might relate to the weaker growth of the microbes used in these raw materials because important flavour compounds of this group are formed from carbohydrates and/or protein through the pyruvate route [18], a growth-associated pathway [65], such as 2,3-butanediol and 3-hydroxybutan-2-one. Notably, pea and faba milk contained less carbohydrates and protein (Table 1).

The observed flavour-related changes included the formation of alcohols and acids from corresponding aldehyde precursors, (ii) the metabolization of terpenes into terpenoid-based derivatives and catabolic intermediates, (iii) the formation of pyruvate-derived volatiles from degraded carbohydrates, (iv) amino acid-derived volatiles from degraded protein, and (v) unsaturated fatty acids from lipids (Table 1). The reduction/oxidation of aldehydes into alcohols and organic acids was detected in all fermentations. The highly abundant aldehyde 1-hexanal, causing undesired bean-like notes already at a low threshold (Table 2), decreased by 14% to even 100%, while 1-hexanol and hexanoic acid were formed (Fig. 3).

Terpenoid-related flavour changes were specifically observed during sunflower seed and faba milk fermentation. They seemed to originate from terpene alkenes, such as α-pinene, camphene, α-, β-terpinene, 3-carene, p-cymene, and d-limonene, found in these two plant milks (Fig. 3). These terpenes contribute to a fruity, woody, terpenic, and nutty flavour (Table 3). The formed alcoholic derivatives have a lower odour threshold (Table 2), resulting in an even stronger aroma [66]. In this regard, it was interesting to see that α-pinene and d-limonene were reduced to myrtenol, pinocarveol, and carveol and further converted to other related ketones and esters (e.g., pinocarvone and carvone). As an example, *S. thermophilus*, *L. lactis*, *L. plantarum*, *L. johnsonii*, and *L. sakei* fermented sunflower seed milk to increase the levels of pinocavenol, mytenol, (-)-mytenol, pinocarvone, and myrtenol acetate. Notably, *L. johnsonii* and *L. plantarum* increased the relative amount of (-)-mytenol, related to a nice minty and woody odour, by approximately 50-fold. Substantial linalool formation was observed in faba milk. The volatile is considered one of the most preferred alcoholic terpinenes with regard to its citrus and floral aromas (Table 2) as well as its antioxidant and antimicrobial activities [67–69]. It was present only in a relatively low amount in unfermented faba milk but increased 1.2- to 13.5-fold by fermentation (Fig. 2). Notably, *L. johnsonii* achieved the highest linalool level among all strains. The increase in linalool in plant materials has been previously reported for *L. paracasei*, *L. plantarum*, and *L. rhamnosus* [46, 70] and is regarded as beneficial with regard to the overall aroma.

### *S. thermophilus *NCC 1326 shows high potential to increase preferred buttery notes in different plant-based milks

Additionally, we observed the formation of volatiles with buttery and creamy notes (3-hydroxybutan-2-one, 2,3-butanedione, 2,3-butanediol, 2,3-pentanedione and their ester derivatives) (Fig. 3) obviously originating from sugars through the pyruvate pathway [18] and transamination of amino acids to α-keto acids and further to aldehydes, alcohols, and acids [26]. Almost all samples contained 2-methylbutanal and 3-methylbutanal (malty, cocoa-like flavour) and/or 2-methyl butanol, 3-methyl butanol, 2-methyl butanoic acid and 3-methyl butanoic acid, which exhibit a fruity, ethereal, nice alcoholic and cheesy note (Fig. 3**, **Table 2). These compounds are known to be derived from the degradation of branched-chain amino acids [26]. Finally, the formation of unsaturated aldehydes (e.g., 2,4-decadienal, 2-octenal) observed during fermentation of oat, pea, and faba milk (Fig. 3) seemed to be related to lipid/fatty acid oxidation [71]. This was not the case for sunflower seed milk, related to its negligible fat level (Table 1).

Strikingly, *S. thermophilus* NCC 1326 was able to increase the buttery note in all four plant milks. This can be regarded as very attractive, given that this note is generally preferred in fermented plant milk products. The highest increase in favoured flavours was observed for fermented oat and sunflower seed milk, but fermented pea and faba milk also contained elevated amounts of these flavours. Cheesy and sour odours were increased in fermented pea milk and faba milk. *L. mesenteroides*, *L. plantarum*, and *L. johnsonii* were most effective in increasing these notes (from 0 to 83%). The formation of cheese-like flavours is challenging in plant-based materials since these materials lack the precursor casein [26]. Therefore, fermentation with the mentioned microbes might support the development of non-dairy cheese alternatives from legume-based plant materials. Such products currently gain huge attention based on increased consumer demand [26, 72].

### A data-driven model allows for bridging compositional volatile data with multisensory perception

**Fig. 6:**
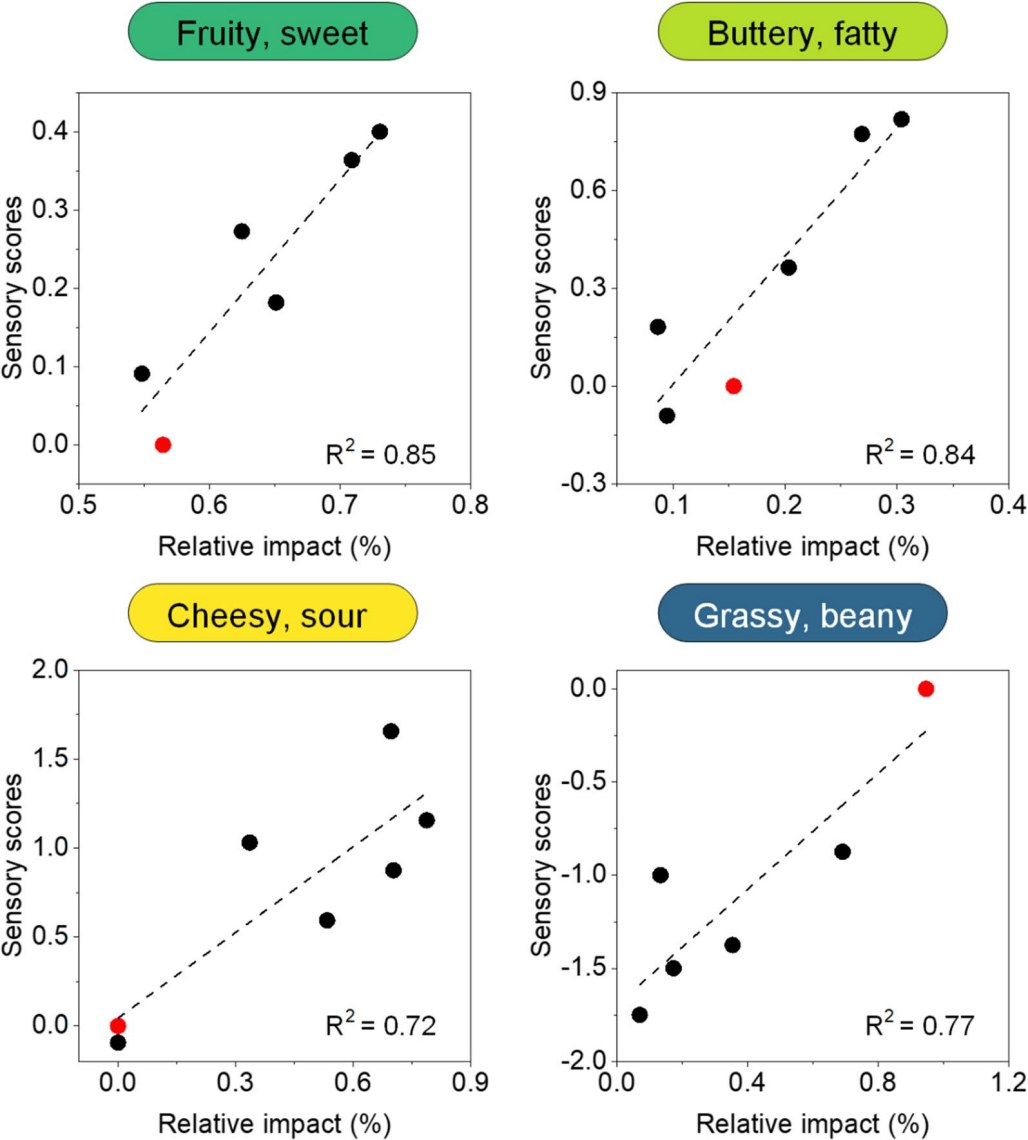
Correlation between predicted perceivable flavour from GC‒MS-based volatile analysis of nonfermented and fermented plant milks and multisensory perception by a panel of untrained participants. The studied milks comprise oat milk (top) and pea milk (bottom) with their most relevant flavour changes. The data shown include (i) fruity, citrus, sweet, melty, and ethereal flavour notes in oat milk (designated fruity, sweet) (top left); (ii) buttery, fatty, waxy, and creamy flavour notes in oat milk (designated buttery, fatty) (top right); (iii) cheesy and sour flavour notes in pea milk (designated cheesy, sour) (bottom left); and (iv) green, grassy, bean-like, and herbal flavour notes in pea milk (designated grassy, green) (bottom right). For each scenario, the unfermented sample is shown in red, while the fermented samples are shown in black

Notably, the predicted increase in flavour notes from the GC‒MS-based analysis matched the perception by the test persons well and even yielded a linear correlation (Fig. 6). The same was true for the removal of grassy/green off-flavours from pea milk, recognized in the sensory test and matching the estimation from compositional analysis. In addition, the formation of cheesy/sour aromas in fermented pea milk revealed a significant correlation between the determined GC‒MS-based odour implication on one hand and the human sensory score on the other hand (Fig. 6). Because the estimation was based on relative amounts of flavour volatiles, which are much easier to assess than absolute values, the workflow appears attractive to be used further. Admittedly, it requires odour threshold data, which might not be available for every compound of interest. Here, we could cover 70% of all analytes with a known flavour note. Hopefully, more threshold data will be added in the future. Generally, predicting human perception by instrumental measurements is quite challenging [73].

In our opinion, the correction and weighing of measurement data by integrating peak areas of individual compounds with flavour threshold, as proposed here, displayed an important step to upgrade the data and bring them closer to real sensory reception. The novel approach opens several promising options. Given its potential, it seems worth to fine-tune the analytical and data processing pipeline and create an expert data base for plant milk-based volatiles. As example, additional measurements with a broader set of external standards could support compound identification and investigate matrix effects, while, on top of the used mass spectral deconvolution approach, sophisticated scoring algorithms appear promising to eventually reduce false positives and negatives [74]. The consideration of signal information from extracted ion chromatograms (EIC) could help to improve quantitative accuracy, particularly for low abundance volatiles [75]. In addition, the involvement of machine learning appears promising for further training and fine-tuning [76, 77], creating a more rapid, repeatable cyclical workflow for the discovery and prediction of flavour formation. In this regard, our approach could support analytical efforts that use, for example, GC‒MS, electronic sensor arrays (e-nose), and chemometric methods to evaluate tea [78], olive oil [79], and tofu products [80]. Furthermore, it would appear interesting to couple the analyses with 1D and 2D GC- coupled olfactometry (sniffing analysis) to confirm the aromatic impact of the selected compounds [81].

## Conclusion

Flavour is one of the most important attributes that consumers consider when selecting food, including plant milk [4]. As plant milk products naturally suffer from undesired flavour, masking undesired and improving desired notes is a key issue in the plant milk alternative market. Here, we investigated a selection of 15 LAB strains from various genera and species regarding their capacity to affect the flavour profile of four types of plant milks of high commercial interest, a cereal-based milk (oat) [82], a seed-based milk (sunflower seed) [7], and two legume-based milks (pea and faba) [83]. Based on GC‒MS volatile analysis and transformation of compositional volatile data into predicted flavour profiles and odour thresholds, we showed that fermentation strongly improved the flavour of the fermented plant milks. A few key conclusions can be drawn at this stage.

The different set-ups created great diversity in aroma profiles, underlining the complexity of the associated flavour metabolism. The observed flavour changes were associated with the nutrient composition of the used plant milk. As an example, terpenoid-based flavours were formed in sunflower seed and faba milk, rich in these precursors, while lipid-related flavours were less prominent due to the lack of fat in the raw material. Given this wide spectrum, a careful selection of specific combinations of milk and strain allowed for the design of specific flavour profiles. Our test set of 60 possible combinations offered specific selections that either yielded a strong buttery flavour, cheesy flavour, or woody flavour, opening new options towards more diverse flavour-tailored plant milk-based products.

Notably, a few strains revealed generally beneficial properties, independent of the plant milk. As an example, *S. thermophilus* NCC 1326 worked as a “butter-maker” in strongly enhancing buttery notes in all plant milks, while *L. mesenteroides* emerged as a “cheese-maker” that widely increased cheesy notes, and *L. johnsonii* proved to be a good “minty/woody-maker”, given its active terpenoid-based flavour metabolism. This includes the discovery that *L. johnsonii* performed well, independent of the plant milk used. Their identification was an important finding, given that the strain selection for plant-based milk fermentation is challenging, given the huge compositional variation between the different plant-based matrices. In this regard, our approach allowed to study the potential of various strains and identify the best candidates according to flavor improvement capability. The highlighted strains and plant milks appear promising to be analysed in more detail in the future. Future studies of the most promising combinations from our screening should then include the monitoring of changes in in nutritional composition, colour, and texture toward a more complete picture of the obtained product properties. In addition, it appears promising to extend our approach also to other plant-based milks.

## Material and methods

### Microorganisms

The microbial strains used in this work were obtained from the Nestle Culture Collection (NCC, Lausanne, Switzerland) (Additional file 2: Table S2). All strains were food-grade approved based on the qualified presumption of safety (QPS) recommendation [84]. They were maintained as frozen stocks in 30% glycerol (v/v) at − 80 °C.

### Strain-specific preculture and main culture media

Depending on individual nutrient requirements, specific media were used for pre-cultivation of the different strains (Additional file 2: Table S2). *Streptococcus* and *Lactococcus* strains were grown in HJL medium, adjusted to a final pH value of 6.5, containing 30 g of tryptone (Becton Dickinson, Franklin Lakes, NJ, USA), 10 g of yeast extract (Becton Dickinson), 10 g of lactose (Sigma‒Aldrich, Steinheim, Germany), 2 g of beef extract (Life Technologies Corporation, Detroit, MI, USA), and 5 g of KH_2_PO_4_ [85]. Strains of *Lacticaseibacillus*, *Lactiplantibacillus*, *Lactilactobacillus*, *Lactobacillus*, *Limosilactobacillus*, and *Leuconostoc* were grown in de-Man‐Rogosa‐Sharpe (MRS) medium containing 52 g of MRS broth (Sigma‒Aldrich) and 1.0 mL of Tween-80 (Sigma‒Aldrich) per litre [86].

### Plant milk medium

Sterilization of the plant milks by ultrahigh temperature (UHT) treatment was performed as described previously [7]. Briefly, a plant milk suspension was prepared by mixing the plant material with deionized water. The suspension was then homogenized and preheated to 75 °C, immediately followed by UHT treatment. Hereby, the prewarmed suspension was heated for 4 s to 143 °C at a flow rate of 30 L h^−1^ and was then efficiently cooled to 4 °C [7]. Finally, the milk was aseptically filled into sterile plastic bottles (2 L) and kept at 4 °C until use. Before fermentation, the sterilized milk was manually homogenized.

### Precultures

Strain-specific settings (medium and temperature) were used to propagate precultures (Additional file 2: Table S2). All strains were grown at 30 °C in 20 mL tubes containing 10 mL of the corresponding growth medium. First, precultures were inoculated from glycerol stocks (200 µL) and were then incubated overnight under a CO_2_-enriched atmosphere (9–13%) (Anaerobic atmosphere generation bags, Merck, Darmstadt, Germany). Afterwards, the first precultures were used as inoculum for the second precultures using an inoculum size of 2% (v/v), which were then grown overnight under the same conditions. An appropriate amount of preculture was centrifuged (5,000 × *g*, 5 min, 4 °C) and resuspended in 150 µL deionized water to serve as inoculum for the main fermentations.

### Plant milk fermentation

Anaerobic fermentations were conducted in 100 mL serum bottles [87, 88] filled with 15 mL of the corresponding plant milk. The cultures were inoculated to a starting cell concentration of 2 × 10^7^ colony formation units (cfu) mL^−1^. The bottles were tightly sealed with an aluminium cap. The strains to be used were known facultative anaerobes. However, we aimed to explore the true anaerobic potential. Therefore, the air in the headspace was replaced by nitrogen [87, 88]. The inoculated plant milks were incubated on a rotary shaker (30 °C, 130 rpm, 80% humidity, 5 cm shaking diameter, Infors, Bottmingen, Switzerland). Three biological replicates were carried out for each fermentation, and non-inoculated fermentations were conducted as a control.

### Quantification of colony-forming units

Colony forming units (cfu) were determined by the plate serial dilution spotting method [18]. Briefly, 1 mL culture samples were sequentially diluted using 0.85% NaCl (w/v) supplemented with 1 g L^−1^ tryptone (Becton Dickinson). For the analysis of *Streptococcus* and *Lactococcus*, samples were spotted on HJL agar. For *Lacticaseibacillus*, *Lactiplantibacillus*, *Lactilactobacillus*, *Lactobacillus*, and *Leuconostoc*, MRS agar was used. All measurements were conducted in duplicate.

### Solid phase microextraction (SPME)

Approximately 5 mL of broth was collected into a 20 mL vial, supplemented with 1 g NaCl, and incubated for 20 min at 40 °C and 400 rpm in the dark (PAL RSI 120, CTC Analytics, Zwingen, Switzerland). Afterwards, the contained volatiles were trapped onto an SPME fibre for another 20 min at 40 °C using two fibre types, divinylbenzene/polydimethylsiloxane (65 µm, 1 cm, DVB/PDMS, Agilent Technologies) and divinylbenzene/carboxen/polydimethylsiloxane (50/30 µm, 1 cm, DVB/CAR/PDMS, Agilent Technologies). A blank sample with water was run before each sequence to ensure the quality of the fibre and check for potential background interference. The fibres were replaced every 50 measurements.

### GC‒MS analysis of volatile flavour compounds

Volatile compounds were analysed by GC‒MS (Agilent Technologies 8890 GC system) as described previously [7, 18]. Prior to split-less injection, the loaded SPME fibre was transferred at 2 cm s^−1^ into the injector port (250 °C) and maintained there for a 3 min desorption interval. After every injection, the fibre was cleaned (20 min, 270 °C). The volatiles were separated on an HP-5MS column (30 m, 250 µm, 0.25 µm, Agilent Technologies) using helium 5.0 as the carrier gas (0.5 mL min^−1^). Chromatograms were recorded by monitoring the total ion current (TIC) over a mass range from 30 to 300 m/z, followed by deconvolution of the obtained signals (MassHunter Workstation Software, Agilent Technologies). For each analyte *i*, measurement and data processing yielded a peak area (*A*_*i*_) that corresponded to the cleaned mass spectrum of the eluting compound, reflecting its absolute abundance. Its relative abundance *a*_*i*_ was then obtained by normalization of *A*_*i*_ to the total peak area (*A*_*total*_) of all volatiles detected in the sample according to Eq. 1.

1 $${\mathrm{a}}_{i} ={A}_{i}/{A}_{total}\times 100$$

### Identification and grouping of volatile compounds

Detected volatiles were identified in three ways. First, the corresponding mass spectra were subjected to a mass spectra library search (NIST/EPA/NIH Mass Spectral Library 08). Compounds were regarded as identified when a match score > 75% to the corresponding library entry was obtained. As the second criterion, we used the retention index (RI), related to a series of commercial n-alkane standards (C8–C40) (Sigma‒Aldrich). Volatile compounds were regarded as identified if the obtained RI matched the reference value. For several analytes, additional structural information was obtained by analysing external standards: hexanal, heptanal, nonanal, benzaldehyde, phenylacetaldehyde, pentanal, octanal, phenylethyl alcohol, 1-pentanol, 1-hexanol, 1-heptanol and 1-octanol, eugenol, acetic acid, α-pinene, and d-limonene. In all cases, these measurements confirmed the identification. It should be noted that sulphur containing compounds appeared to present as well but exhibited low abundance, low signal quality and insufficient match scores so that they were not considered further. The identified volatiles were categorized chemically (assigned to the chemical groups) and functionally (assigned to flavour groups). On the one hand, chemical groups distinguished between aldehydes, alcohols, ketones, organic acids, esters, furans, alkanes, alkenes, and others. To evaluate the abundance of such a chemical group within the overall flavour profile, the individual peak areas of all detected analytes attributed to this chemical group were summed (Eq. 2), with g being the attribute. Likewise, the relative abundance of such a chemical group within the overall profile was obtained (Eq. 3).

2 $${A}_{g}=\sum {A}_{i}$$

3 $${\mathrm{a}}_{g} ={A}_{g}/{A}_{total}\times 100$$

On the other hand, flavour groups considered molecules of similar flavour such as (1) fruity, sweet, ethereal; (2) floral (3) buttery, fatty, creamy, waxy; (4) cheesy, sour; (5) nutty, woody, toasted cocoa, terpenic, balsamic, camphoraceous; (6) green, grassy, bean-like, herbal; (7) earthy, mushroom-like; and (8) pungent, spicy, sharp, phenolic. The aroma perception of the volatiles was collected from the literature (Table 3**, **Additional file 2: Table S7). Compounds without aroma descriptions were classified as unknown and not considered further. The evaluation of the abundance of a flavour group within the overall flavour profile is demonstrated in Sect. 2.10.

To evaluate the abundance of such a flavour group within the overall flavour profile, the individual peak areas of all detected analytes that were attributed to this flavour were summed (Eq. 4), with *g* being the attribute, e.g., fruity, cheesy, or other. Likewise, the relative abundance of such a flavour group within the overall profile was obtained (Eq. 5).

4 $${A}_{g}=\sum {A}_{i}$$

5 $${\mathrm{a}}_{g} ={A}_{g}/{A}_{total}\times 100$$

### Functional prediction of flavour profiles based on odour thresholds

To assess the relative impact of the different volatiles within a sample on the overall flavour note, odour threshold values of the individual compounds in air were considered. The odour thresholds were collected from previous studies (Table 3, Additional file 2: Table S8). In cases where literature data had been collected in water and not air, a correction was needed. In short, odour thresholds in water (C*_i, aqueous_, µg kg^−1^) were converted into odour thresholds in air (C*_i, air,_ µg L^−1^) (Eq. 6), including the following parameters: the air/aqueous partition coefficient (K_i_), the density of water ($\rho$= 0.997 kg L^−1^), the ideal gas volume (V_m_ = 24.77 L mol^−1^), and the molecular weight of the compound (*M*_*i*_, g mol^−1^).

6 $${C}_{ i, air}^{*}={K}_{i}\times \rho \times {{V}_{m}\times C}_{ i, aqeous}^{*}/{M}_{i}\times 1000$$

The partition coefficient was acquired from previous work [89] or estimated by the EPI Suite software (V 4.11). (Additional file 2: Table S7).

The calculation yielded individual odour thresholds in air at which each of the compounds could be sensed, which ranged from low (0.01 ppbv) to high (1.27 × 10^6^ ppbv). The least recognizable compound among all detected compounds in this study was 2-ethyl-furan. Its odour threshold (C^*^_2-ethyl-furan, air_) was used as a reference to obtain relative odour thresholds in air for all other analytes (c^*^_i, air_) (Eq. 7). These values described how easily a compound could be sensed in relation to the reference.

7 $${c}_{ i, air}^{*}={C}_{ i, air}^{*}/{C}_{ 2-\mathrm{ethyl}-\mathrm{furan}, air}^{*}\times 100$$

Then, for each compound, the relative abundance *a*_*i*_ was considered to quantify its impact on the flavour within a mixture, named relative odour activity here (o_i_) (Eq. 8).

8 $${\mathrm{o}}_{i} ={a}_{i}/{c}_{ i, air}^{*}$$

Summing up the odour activity values for molecules that belonged to the same flavour group allowed to generally extract the impact of a certain type of flavour (*o*_*g*_), as shown above (Eq. 9). The comparison of the different notes then provided a rough estimate of the expected overall flavour note.

9 $${o}_{g}=\sum {o}_{i}$$

Estimation of sunflower seed milk protein digestibility and score. PCA and HCA were performed using SPSS (version 24.0), including weighing using unit variance and zero-mean normalization scaling, respectively [90]. Moreover, for PCA, growth was transformed to fold-change of colony forming units (cfu mL^−1^).

### Sensory evaluation

To investigate the validity of the GC‒MS-based functional flavour assignment, flavour changes were evaluated in sensory tests. Fermented and unfermented plant milk samples were pasteurized for 2 min at 71 °C in a water bath before being quickly cooled on ice to room temperature before evaluation. An untrained panel of 8 to 10 participants was asked to evaluate the difference between fermented samples and an unfermented control for specific flavour notes: (i) fruity/sweet, (ii) buttery/fatty, (iii) cheesy/sour, and (iv) grassy/green. If differences were noted, participants noted the intensity perceived change using a scale from -3 (strongly reduced) to 3 (strongly increased) in the question format. Finally, the predicted relative impact of different flavour groups (see above) was compared against the average sensory score.

### Data processing and statistical analysis

All results displayed in Figures and Tables are shown as the mean values ± standard errors. Statistical evaluation of the data was conducted by one-way analysis of variance (ANOVA). Statistical analyses were performed by using SPSS (version 24.0).

## Supplementary Information

**Additional file 1: **GC-MS volatile data of the unfermented and fermented plant milk.

**Additional file 2: Table S1.** Effect of DVB/PDMS and DVB/CWR/PDMS fibres on volatile compound extraction from oat milk, sunflower seed milk, pea milk, and faba milk. **Table S2.** Strain specific pre-culture conditions. Mann‐Rogosa‐Sharpe medium (MRS) and HJL broth was used. **Table S3.** Loading plot of unfermented and fermented oat milk. **Table S4.** Loading plot of unfermented and fermented sunflower seed milk. **Table S5.** Loading plot of unfermented and fermented pea milk. **Table S6.** Loading plot of unfermented and fermented faba milk. **Table S7.** Identified volatile compounds in unfermented and fermented plant-based milks, aroma attributes, odor groups, and the odor threshold in air. **Table S8.** Calculation of the odor threshold (OT) of the volatiles in air (ppbv).

## Data Availability

The dataset(s) supporting the conclusions of this article are all included within the article.
