# Supplementary material for: Flavour by design: food-grade lactic acid bacteria improve the volatile aroma spectrum of oat milk, sunflower seed milk, pea milk, and faba milk towards improved flavour and sensory perception
Source: Microb Cell Fact. 2023 Jul 21;22:133. doi: 10.1186/s12934-023-02147-6 (PMC10362582; doi:10.1186/s12934-023-02147-6)
Supplement: Supplementary file 2 — Additional file 2: Table S1. Effect of DVB/PDMS and DVB/CWR/PDMS fibres on volatile compound extraction from oat milk, sunflower seed milk, pea milk, and faba milk. Table S2. Strain specific pre-culture conditions. Mann‐Rogosa‐Sharpe medium (MRS) and HJL broth was used. Table S3. Loading plot of unfermented and fermented oat milk. Table S4. Loading plot of unfermented and fermented sunflower seed milk. Table S5. Loading plot of unfermented and fermented pea milk. Table S6. Loading plot of unfermented and fermented faba milk. Table S7. Identified volatile compounds in unfermented and fermented plant-based milks, aroma attributes, odor groups, and the odor threshold in air. Table S8. Calculation of the odor threshold (OT) of the volatiles in air (ppbv). [file 12934_2023_2147_MOESM2_ESM.docx]

**Additional file 2 to**

**Flavour by design: Food-grade lactic acid bacteria improve the volatile aroma spectrum of oat milk, sunflower seed milk, pea milk and faba milk towards improved flavour and sensory perception**

Muzi Tangyu^1^, Michel Fritz^1^, Jan Patrick Tan^2^, Lijuan Ye^2^, Christoph J. Bolten^2,3^, Biljana Bogicevic^2^, and Christoph Wittmann^1#^

^1^Institute of Systems Biotechnology, Saarland University, Saarbrücken, Germany

^2^ Nestlé Research Center, Lausanne, Switzerland

Contact information

[muzi.tangyu@uni-saarland.de](mailto:muzi.tangyu@uni-saarland.de)

[christoph.wittmann@uni-saarland.de](mailto:christoph.wittmann@uni-saarland.de)

^#^ Phone/FAX: +49 681 302 71970/71972, e-mail: [christoph.wittmann@uni-saarland.de](mailto:christoph.wittmann@uni-saarland.de)

**Table S1.** Effect of DVB/PDMS and DVB/CWR/PDMS fibres on volatile compound extraction from oat milk, sunflower seed milk, pea milk, and faba milk.

|  | Oat milk | | Sunflower seed milk | | Pea milk | | Faba milk | |
| --- | --- | --- | --- | --- | --- | --- | --- | --- |
|  | PDMS/DVB | DVB/CAR/PDMS | PDMS/DVB | DVB/CAR/PDMS | PDMS/DVB | DVB/CAR/PDMS | PDMS/DVB | DVB/CAR/PDMS |
| Aldehydes | 10 | 12 | 5 | 7 | 10 | 12 | 10 | 12 |
| Alcohols | 6 | 7 | 11 | 13 | 5 | 8 | 4 | 7 |
| Ketones | 4 | 6 | 3 | 5 | 3 | 3 | 2 | 2 |
| Organic acids | 2 | 3 | 1 | 1 | 1 | 1 | 1 | 2 |
| Esters | 1 | 3 | 1 | 3 | 0 | 0 | 0 | 0 |
| Furans | 2 | 3 | 1 | 2 | 1 | 1 | 1 | 1 |
| Alkanes | 2 | 2 | 5 | 3 | 3 | 0 | 3 | 0 |
| Alkenes | 3 | 4 | 7 | 9 | 1 | 0 | 0 | 2 |
| Others | 3 | 4 | 3 | 7 | 3 | 3 | 2 | 2 |
| Total peak number | 33 | 44 | 37 | 50 | 27 | 28 | 23 | 28 |
| Total peas area (1 × 10^7^) | - 1. ± 0.8 | 5.3 ± 0.7 | 5.0 ± 0.4 | 5.6 ± 1.7 | 3.2 ± 0.4 | 3.4 ± 0.6 | 1.6 ± 0.2 | 1.6 ± 0.4 |

*PDMS, polydimethylsiloxane; CAR, carboxen; DVB, divinylbenzene.

Solid-phase microextraction (SPME) has been widely used for analysis of plant-based materials due to its fast and simple operation and a high sensitivity, which enables qualitative and quantitative analysis of flavors even at low concentration [[1](#_ENREF_1), [2](#_ENREF_2)]. Notably, the efficiency of SPME strongly depends on the geometry and the material of the fiber coating [[2](#_ENREF_2)]. Fibers, coated with bi-polar materials such as DVB/PDMS and DVB/CAR/PDMS are types often used for the application of plant-based milks and other plant-based foods [[3-7](#_ENREF_3)]. Therefore, to evaluate volatile compound extraction, those materials were tested for unfermented plant milks. The impact of the different fibers on the extracted number of volatile compounds is shown in **Table 1**. In general, both fibers well extracted aldehydes, alcohols, ketones, furans, organic acids, esters, alkanes, and alkenes from the tested plant milks. However, the fibers behaved differently in their ability to adsorb key flavor compounds. The DVB/CAR/PDMS fiber absorbed between 20 and 50 volatiles from oat milk, sunflower seed milk, pea milk, and faba milk respectively, while the DVB/PDMS fiber trapped 25-34 volatiles, respectively. The triple DVB/CAR/PDMS fiber was more efficient in extracting key flavor compounds, especially aldehydes, alcohols, and ketones, accounting for the most important flavor groups of unfermented plant milks [[3](#_ENREF_3), [5](#_ENREF_5), [7](#_ENREF_7), [8](#_ENREF_8)]. As the main aim of this study was to qualitatively identify volatile compounds during the fermentation, the DVB/CAR/PDMS fiber was chosen for further studies.

**Table S2.** **Strain specific pre-culture conditions.** Mann‐Rogosa‐Sharpe medium (MRS) and HJL broth was used.

| **Strains** | **Medium** | **Temperature (°C)** |
| --- | --- | --- |
| [***Streptococcaceae***](https://www.google.com/search?rlz=1C1ONGR_zh-CNDE931DE931&sxsrf=ALiCzsYVOKr412x2EwYkGBeUh6fDUM-oAw:1654341271208&q=Streptococcaceae&stick=H4sIAAAAAAAAAONgVuLUz9U3SDIzKylaxCoQXFKUWlCSn5yfnJyYnJqYCgC24WDmIAAAAA&sa=X&ved=2ahUKEwiT17q-1ZP4AhWph_0HHXO-ChoQmxMoAXoECFoQAw) |  |  |
| *Streptococcus thermophilus* NCC 1326 | HJL | 37 |
| *S. thermophilus* NCC 1988 | HJL | 37 |
| *S. thermophilus* NCC 2019 | HJL | 37 |
| *S. thermophilus* NCC 2059 | HJL | 37 |
| *Lactococcus lactis* NCC 2180 | HJL | 30 |
| *L. lactis* NCC 2242 | HJL | 30 |
| ***Lactobacillaceae*** |  |  |
| *Lacticaseibacillus rhamnosus* NCC 2891 | MRS | 37 |
| *L. rhamnosus* NCC 4007 | MRS | 37 |
| *L. paracasei* subsp. *paracasei* NCC 2511 | MRS | 30 |
| *Lactiplantibacillus plantarum* NCC 2988 | MRS | 30 |
| *Lactilactobacillus sakei* NCC 1692 | MRS | 30 |
| *Lactobacillus johnsonii* NCC 533 | MRS | 37 |
| *Lactobacillus helveticus* NCC 1276 | MRS | 30 |
| *Limosilactobacillus fermentum* NCC 660 | MRS | 37 |
| *Leuconostoc mesenteroides* NCC 2832 | MRS | 30 |

**Table S3.** Loading plot of unfermented and fermented oat milk.

|  | PC1 (23.11%) | PC2 (18.49%) | PC3 (14.68%) |
| --- | --- | --- | --- |
| 3-Methyl-butanal | -0.59 | 1.07 | 0.80 |
| 2-Methyl-butanal | -0.53 | 1.11 | 0.91 |
| Hexanal | -0.57 | 1.71 | 1.69 |
| 2,4-Heptadienal | 0.07 | -0.39 | 0.73 |
| 2-Heptenal | 0.15 | 1.06 | -0.09 |
| Heptanal | 0.09 | 0.88 | 1.14 |
| Benzaldehyde | -0.65 | 0.82 | 1.77 |
| 2-Octenal | 0.77 | 1.69 | -0.40 |
| Phenylacetaldehyde | 0.00 | 0.00 | 0.00 |
| 4-Ethyl-benzaldehyde | 0.37 | 0.61 | 0.68 |
| 2-Nonenal | 0.38 | 1.15 | 0.35 |
| Nonanal | -1.05 | 0.97 | 0.91 |
| 2,4-Decadienal | 1.30 | 1.23 | -0.89 |
| Decanal | -0.23 | -2.62 | 0.15 |
| [S,S]-2,3-Butanediol | -0.88 | -1.45 | -0.71 |
| 3-Methyl-1-butanol | -1.17 | 0.77 | 0.11 |
| 2-Methyl-1-butanol | -1.08 | -0.01 | 0.91 |
| 1-Pentanol | 1.14 | 0.95 | -0.03 |
| 1-Hexanol | 1.68 | -0.27 | 0.23 |
| 2-Heptanol | -0.23 | -2.62 | 0.15 |
| 1-Heptanol | 0.78 | -2.21 | 0.65 |
| 2-Methyl-3-hexanol | -1.17 | 0.77 | 0.11 |
| 1-Octen-3-ol | 1.01 | 0.04 | 0.83 |
| 2-Octen-1-ol | 0.31 | -1.90 | 1.39 |
| 1-Octanol | 0.97 | -1.55 | 1.49 |
| 1-Nonanol | 0.86 | -1.00 | 0.63 |
| (-)-Myrtenol | 0.11 | 1.24 | 0.45 |
| Terpinen-4-ol | -0.59 | 1.07 | 0.80 |
| 3-Ethyl-4-nonanol | 0.11 | 1.24 | 0.45 |
| Methyl vinyl ketone | -0.57 | 1.71 | 1.69 |
| 2,3-Butanedione | 0.90 | 0.72 | -1.41 |
| 3-Hydroxy-2-butanone | 0.54 | 1.16 | -0.59 |
| 2,3-Pentanedione | 0.09 | 0.88 | 1.14 |
| 2-Hexanone | 1.02 | -1.43 | -0.58 |
| 2,3-Heptanedione | 0.80 | 0.13 | -1.07 |
| 2-Heptanone | 0.26 | 2.00 | -0.26 |
| 1-(2-Furanyl)-1-propanone | -0.54 | 0.96 | 1.69 |
| 3,5-Octadien-2-one | 1.26 | 0.59 | 1.00 |
| 6-Methyl-5-hepten-2-one | -0.24 | 0.91 | 0.99 |
| 2-Nonanone | 1.04 | 0.92 | 0.73 |
| 5,6-Dehydrocamphor | 1.60 | -0.62 | 0.10 |
| Pinocarvone | 1.04 | -1.72 | 0.90 |
| Acetic acid | -0.92 | 0.14 | 0.33 |
| 3-Methyl-butanoic acid | -1.39 | -0.43 | 0.47 |
| 2-Methyl-butanoic acid | 1.21 | -1.64 | 0.59 |
| Pentanoic acid | 0.07 | -0.39 | 0.73 |
| Hexanoic acid | -0.60 | -0.67 | 1.04 |
| Octanoic Acid | 0.89 | 0.76 | 0.87 |
| Nonanoic acid | 1.36 | 0.65 | -0.82 |
| Ethyl Acetate | -0.30 | 1.08 | 0.47 |
| Ethyl lactate | 0.29 | -2.08 | 0.61 |
| Ethyl 2-methylbutyrate | 1.07 | 1.69 | -0.24 |
| Hexyl acetate | 0.91 | -0.32 | 2.00 |
| Verbenyl acetate | 1.07 | 1.69 | -0.24 |
| Bornyl acetate | 0.91 | -0.32 | 2.00 |
| 2-n-Butyl furan | -0.31 | -2.62 | 0.07 |
| 2-Pentyl-furan | 0.91 | -0.50 | 1.97 |
| 2-n-Heptylfuran | -0.09 | 0.19 | 1.53 |
| Dodecane | 1.04 | 0.92 | 0.73 |
| Tridecane | 1.60 | -0.62 | 0.10 |
| a -Pinene | 1.04 | -1.72 | 0.90 |
| β-Terpinene | 0.90 | 0.72 | -1.41 |
| 3-Carene | 1.63 | -0.24 | 1.04 |
| o-Cymene | 1.21 | -1.64 | 0.59 |
| D-Limonene | 1.34 | 0.24 | 0.68 |
| Alloocimene | 1.22 | 0.11 | -1.46 |
| 2,4-Dimethyl-1-decene | 0.26 | 2.00 | -0.26 |
| β-Gurjunene | -0.54 | 0.96 | 1.69 |
| Dimethyl ether | -0.88 | -1.72 | -0.48 |
| 4-Ethenyl-1,2-dimethyl-benzene | -0.24 | 0.91 | 0.99 |
| 4-Methyl-2-propylphenol | 0.19 | -0.26 | 1.55 |
| Unknown4 | 1.65 | 0.75 | -0.50 |
| Unknown5 | 1.65 | 0.75 | -0.50 |
| Unknown6 | -0.88 | -1.72 | -0.48 |
| Growth | -0.73 | -1.22 | 0.19 |

**Table S4. Loading plot of unfermented and fermented sunflower seed milk.**

|  | PC1 (38.98%) | PC2 (15.81%) | PC3 (10.73%) |
| --- | --- | --- | --- |
| 3-Methyl-butanal | -0.76 | -0.07 | 1.85 |
| 2-Methyl-butanal | -0.84 | -0.12 | 1.75 |
| Hexanal | -0.63 | -0.02 | 2.57 |
| 2,4-Heptadienal | 0.00 | 0.00 | 0.00 |
| 2-Heptenal | 1.68 | 1.02 | -0.89 |
| Heptanal | -0.60 | -0.18 | 2.44 |
| Benzaldehyde | 0.32 | -0.80 | 1.61 |
| 2-Octenal | -0.05 | -0.36 | -1.82 |
| Phenylacetaldehyde | -0.56 | -0.30 | 1.83 |
| 4-Ethyl-benzaldehyde | 2.41 | -0.24 | 0.66 |
| 2-Nonenal | 0.23 | 1.46 | -0.27 |
| Nonanal | -0.82 | 0.31 | 2.29 |
| 2,4-Decadienal | 0.86 | 1.37 | -1.37 |
| Decanal | 0.00 | 0.00 | 0.00 |
| [S,S]-2,3-Butanediol | -0.05 | -0.36 | -1.82 |
| 3-Methyl-2-buten-1-ol | 2.41 | -0.25 | 0.66 |
| 3-Methyl-1-butanol | 2.41 | -0.24 | 0.66 |
| 2-Methyl-1-butanol | 0.23 | 1.46 | -0.27 |
| 1-Pentanol | 0.06 | 1.76 | 0.18 |
| 1-Hexanol | 0.86 | 1.37 | -1.37 |
| 2-Heptanol | 1.69 | -0.49 | 0.62 |
| 1-Heptanol | 1.08 | 0.62 | -1.78 |
| Benzyl alcohol | 2.41 | -0.27 | 0.67 |
| 2-Methyl-3-hexanol | 1.11 | 0.89 | -0.84 |
| Phenylethyl alcohol | 2.40 | -0.29 | 0.68 |
| 1-Octen-3-ol | 1.16 | 0.40 | 1.38 |
| 2-Octen-1-ol | 1.69 | -0.49 | 0.62 |
| 3-Octanol | 1.84 | 0.71 | -0.07 |
| 2-Ethyl-1-hexanol | 2.41 | 0.12 | 0.72 |
| 1-Octanol | 1.11 | 0.89 | -0.84 |
| 1-Nonanol | -0.85 | -0.98 | 0.13 |
| Eugenol | 2.41 | -0.26 | 0.67 |
| Cherry propanol | 1.84 | 0.71 | -0.07 |
| laevo-Pinocarveol | 2.40 | -0.30 | 0.68 |
| cis-Verbenol | 2.33 | -0.06 | 0.67 |
| (-)-Myrtenol | 2.41 | -0.26 | 0.67 |
| Myrtenol | 2.41 | -0.11 | 0.74 |
| Terpinen-4-ol | 2.32 | 0.50 | -0.02 |
| 3-Ethyl-4-nonanol | 0.94 | -0.90 | 0.42 |
| Methyl vinyl ketone | -0.63 | -0.02 | 2.57 |
| 2,3-Butanedione | 2.08 | -0.24 | 0.49 |
| 3-Hydroxy-2-butanone | 0.70 | 1.04 | -1.56 |
| 2,3-Pentanedione | -0.60 | -0.18 | 2.44 |
| 2-Hexanone | 0.03 | 0.35 | -0.99 |
| 3,6-Heptanedione | 1.69 | -0.49 | 0.62 |
| 2-Heptanone | 0.20 | 1.41 | 1.04 |
| 1-(2-Furanyl)-1-propanone | -0.45 | 0.23 | 0.58 |
| 3,5-Octadien-2-one | -0.82 | 0.31 | 2.29 |
| Isoacetovanillone | 1.26 | -0.82 | -0.62 |
| 2-Nonanone | 0.29 | 1.61 | -0.51 |
| 5,6-Dehydrocamphor | 1.86 | 0.03 | -0.70 |
| Pinocarvone | 2.15 | 0.70 | 0.62 |
| D-Verbenone | 1.38 | 0.40 | 0.15 |
| Acetic acid | -0.21 | 1.49 | -0.76 |
| 3-Methyl-butanoic acid | -1.59 | 0.67 | 0.43 |
| 2-Methyl-butanoic acid | 2.04 | -0.06 | -0.30 |
| Pentanoic acid | -1.38 | -0.24 | 1.37 |
| Hexanoic acid | 1.68 | 1.02 | -0.89 |
| Octanoic Acid | -0.03 | 0.33 | -2.08 |
| Nonanoic acid | 1.85 | 0.17 | -1.42 |
| Ethyl lactate | 0.40 | -1.09 | -0.98 |
| Verbenyl acetate | 2.41 | 0.35 | 0.44 |
| Bornyl acetate | 2.41 | -0.26 | 0.67 |
| Epoxy-.alpha.-terpenyl acetate | 2.44 | -0.11 | 0.67 |
| 2-Ethyl-furan | 2.41 | 0.35 | 0.44 |
| 2-Ethyl-5-methylfuran | -0.64 | 0.51 | -0.13 |
| 2-Acetyl-5-methylfuran | 0.53 | 1.53 | 0.02 |
| 2-Pentyl-furan | -1.01 | 1.23 | 0.39 |
| Undecane | 1.26 | -0.82 | -0.62 |
| Dodecane | -2.18 | -0.18 | -0.65 |
| Tridecane | 1.32 | -0.47 | 0.07 |
| a-Pinene | -1.59 | 0.97 | 0.62 |
| Camphene | -1.42 | 1.06 | 0.77 |
| β-Terpinene | -1.50 | 1.08 | 0.69 |
| 3-Carene | -1.59 | 0.67 | 0.43 |
| o-Cymene | 2.44 | 0.24 | 0.37 |
| D-Limonene | -1.38 | -0.24 | 1.37 |
| γ-Terpinene | 2.22 | -0.03 | 0.76 |
| α-Thujene | 2.40 | -0.21 | 0.64 |
| β-Thujene | 2.25 | 0.67 | 0.18 |
| 2,4-Dimethyl-1-decene | 0.20 | 1.41 | 1.04 |
| β-Gurjunene | 0.40 | -1.09 | -0.98 |
| Dimethyl ether | -0.28 | -0.55 | -2.21 |
| 4-Ethenyl-1,2-dimethyl-benzene | 1.56 | -0.22 | 0.19 |
| α-Limonene diepoxide | -2.02 | -0.07 | -0.14 |
| Unknown2 | -2.29 | 0.42 | -0.20 |
| Unknown3 | -0.59 | 1.54 | 1.26 |
| Unknown4 | 2.41 | 0.12 | 0.72 |
| Unknown5 | 2.15 | 0.70 | 0.62 |
| Unknown6 | 0.76 | 0.15 | -0.63 |
| Unknown7 | -0.59 | 1.54 | 1.26 |
| Unknown8 | -0.28 | -0.55 | -2.21 |
| Growth | 0.83 | -0.27 | -1.57 |

**Table S5. Loading plot of unfermented and fermented pea milk.**

|  | PC1 (29.66%) | PC2 (22.59%) | PC3 (10.33%) |
| --- | --- | --- | --- |
| 3-Methyl-butanal | -1.74 | 0.66 | -0.24 |
| 2-Methyl-butanal | 0.07 | 1.54 | 1.52 |
| Hexanal | 0.94 | -0.63 | -1.28 |
| 2,4-Heptadienal | 1.63 | 0.22 | 0.66 |
| 2-Heptenal | 1.33 | -0.53 | -0.19 |
| Heptanal | 0.89 | -0.18 | -1.12 |
| Benzaldehyde | 0.39 | -0.91 | 2.08 |
| 2-Octenal | 1.75 | 0.06 | -0.15 |
| Phenylacetaldehyde | 1.13 | -0.57 | -1.57 |
| 4-Ethyl-benzaldehyde | 1.31 | -0.60 | -0.28 |
| 2-Nonenal | 0.00 | 0.00 | 0.00 |
| Nonanal | 1.66 | 0.06 | -0.52 |
| 2,4-Decadienal | 1.75 | 0.04 | 0.27 |
| Decanal | 1.37 | -0.24 | 0.85 |
| [S,S]-2,3-Butanediol | 0.60 | 1.76 | -0.72 |
| 3-Methyl-2-buten-1-ol | 0.34 | 1.57 | 0.77 |
| 1-Pentanol | 0.97 | 0.69 | -0.16 |
| 1-Hexanol | 0.90 | 1.59 | 0.06 |
| 2-Heptanol | -0.94 | 1.18 | -1.71 |
| 1-Heptanol | 0.60 | 1.76 | -0.72 |
| Benzyl alcohol | 0.34 | 1.57 | 0.77 |
| 2-Methyl-3-hexanol | 1.10 | 1.30 | 0.32 |
| 1-Octen-3-ol | 1.38 | 0.59 | -0.48 |
| 2-Octen-1-ol | 0.20 | 1.86 | -0.52 |
| 2-Ethyl-1-hexanol | 0.97 | 0.77 | -1.45 |
| 1-Octanol | 1.10 | 1.30 | 0.32 |
| 1-Nonanol | -0.53 | -0.53 | 1.71 |
| (-)-Myrtenol | 1.21 | -0.19 | 1.25 |
| Myrtenol | 0.98 | 0.88 | 0.93 |
| Linalool | -1.10 | -0.71 | 2.11 |
| 3-Ethyl-4-nonanol | 1.21 | -0.19 | 1.25 |
| Methyl vinyl ketone | 0.94 | -0.63 | -1.28 |
| 2,3-Butanedione | 1.28 | 0.69 | 0.49 |
| 3-Hydroxy-2-butanone | 1.33 | -0.53 | -0.19 |
| 2,3-Pentanedione | 1.28 | 0.72 | 0.35 |
| 2-Hexanone | 0.39 | -0.91 | 2.08 |
| 2,3-Heptanedione | 1.75 | 0.06 | -0.15 |
| 3,6-Heptanedione | 1.13 | -0.57 | -1.57 |
| 2-Heptanone | 0.78 | -0.74 | 1.38 |
| 1-(2-Furanyl)-1-propanone | 0.03 | -1.50 | -0.14 |
| 3,5-Octadien-2-one | 1.47 | -0.42 | 0.36 |
| 6-Methyl-5-hepten-2-one | -0.57 | 0.14 | 2.80 |
| 5-Methyl-3-hepten-2-one | 1.48 | 0.06 | -1.05 |
| 3-Octanone | 1.37 | -0.24 | 0.85 |
| Isoacetovanillone | 1.66 | 0.42 | 0.80 |
| Acetic acid | -1.74 | 0.66 | -0.24 |
| 3-Methyl-butanoic acid | 0.07 | 1.54 | 1.52 |
| 2-Methyl-butanoic acid | 0.23 | 1.14 | 2.36 |
| Hexanoic acid | -0.18 | 1.34 | 2.12 |
| Octanoic Acid | -0.21 | 1.83 | 0.22 |
| Nonanoic acid | -0.87 | 1.26 | 0.21 |
| 2-Ethyl-furan | -0.44 | -0.66 | 0.26 |
| 2-(1-Pentenyl)-furan | -0.97 | 1.02 | -0.97 |
| 2-Pentyl-furan | 1.12 | -0.71 | -0.06 |
| Undecane | -0.97 | 1.02 | -0.97 |
| Dodecane | 1.12 | -0.71 | -0.06 |
| β-Terpinene | 1.28 | 0.69 | 0.49 |
| o-Cymene | 1.28 | 0.72 | 0.35 |
| 2,4-Dimethyl-1-decene | 0.78 | -0.74 | 1.38 |
| β-Gurjunene | 0.03 | -1.50 | -0.14 |
| Dimethyl ether | -1.10 | 1.16 | -1.43 |
| 4-Ethenyl-1,2-dimethyl-benzene | -0.57 | 0.14 | 2.80 |
| 4-Methyl-2-propylphenol | 1.48 | 0.06 | -1.05 |
| Unknown1 | -1.10 | -0.71 | 2.11 |
| Unknown4 | 0.98 | 0.88 | 0.93 |
| Growth | -0.98 | 0.99 | -0.70 |

**Table S6. Loading plot of unfermented and fermented faba milk.**

|  | PC1 (21.66%) | PC2 (18.81%) | PC3 (15.51%) |
| --- | --- | --- | --- |
| 3-Methyl-butanal | 1.34 | -2.24 | 0.99 |
| 2-Methyl-butanal | 2.53 | -0.94 | 0.60 |
| Hexanal | 1.09 | -2.26 | 1.18 |
| 2,4-Heptadienal | 0.00 | 0.00 | 0.00 |
| 2-Heptenal | -1.73 | 0.47 | 0.99 |
| Heptanal | 1.34 | -2.24 | 0.99 |
| Benzaldehyde | 1.65 | -0.85 | -1.09 |
| 2-Octenal | 0.09 | -1.35 | -0.29 |
| Phenylacetaldehyde | 2.50 | -0.53 | 0.96 |
| 4-Ethyl-benzaldehyde | 1.80 | 1.56 | -1.01 |
| 2-Nonenal | -2.09 | 0.20 | 0.42 |
| Nonanal | 1.43 | -2.40 | 0.91 |
| 2,4-Decadienal | -1.65 | 0.19 | 0.54 |
| Decanal | 2.27 | -0.32 | 0.62 |
| [S,S]-2,3-Butanediol | 0.59 | 2.39 | 0.94 |
| 3-Methyl-2-buten-1-ol | 1.41 | 1.46 | -1.39 |
| 3-Methyl-1-butanol | 1.80 | 1.56 | -1.01 |
| 2-Methyl-1-butanol | -2.09 | 0.20 | 0.42 |
| 1-Pentanol | 0.96 | 2.26 | 1.07 |
| 1-Hexanol | -0.84 | 2.41 | 1.08 |
| 2-Heptanol | 0.10 | 1.75 | 0.45 |
| 1-Heptanol | 0.59 | 2.39 | 0.94 |
| Benzyl alcohol | 1.41 | 1.46 | -1.39 |
| 2-Methyl-3-hexanol | 1.37 | 1.16 | 0.48 |
| Phenylethyl alcohol | 0.09 | 0.24 | -1.48 |
| 1-Octen-3-ol | 0.89 | 2.45 | 0.18 |
| 2-Octen-1-ol | -0.24 | 2.06 | 0.80 |
| 3-Octanol | 0.01 | -0.35 | -0.81 |
| 2-Ethyl-1-hexanol | 1.93 | 1.45 | -0.76 |
| 1-Octanol | 1.37 | 1.16 | 0.48 |
| 1-Nonanol | -1.90 | 1.43 | 0.73 |
| Eugenol | 1.49 | 0.93 | -1.35 |
| laevo-Pinocarveol | 0.20 | -0.02 | -1.12 |
| Linalool | 0.20 | -0.02 | -1.12 |
| Terpinen-4-ol | 1.34 | -2.24 | 0.99 |
| 3-Ethyl-4-nonanol | 2.53 | -0.94 | 0.60 |
| Methyl vinyl ketone | 1.34 | -2.24 | 0.99 |
| 2,3-Butanedione | -1.43 | -0.31 | -0.23 |
| 3-Hydroxy-2-butanone | -1.89 | -0.01 | 0.41 |
| 2,3-Pentanedione | 1.34 | -2.24 | 0.99 |
| 2-Hexanone | 1.65 | -0.85 | -1.09 |
| 2,3-Heptanedione | 0.09 | -1.35 | -0.29 |
| 3,6-Heptanedione | 2.50 | -0.53 | 0.96 |
| 2-Heptanone | 0.83 | -0.24 | 0.26 |
| 3,5-Octadien-2-one | 1.20 | 0.38 | -0.16 |
| 6-Methyl-5-hepten-2-one | -1.65 | 0.19 | 0.54 |
| 5-Methyl-3-hepten-2-one | 0.22 | 0.94 | 1.71 |
| 3-Octanone | -0.22 | -0.43 | -1.43 |
| 2-Nonanone | -1.11 | -0.33 | 0.24 |
| Acetic acid | 1.45 | 1.20 | -0.62 |
| 3-Methyl-butanoic acid | -0.01 | 2.24 | 0.41 |
| 2-Methyl-butanoic acid | 2.34 | 0.89 | 0.57 |
| Nonanoic acid | 0.53 | 1.35 | -0.90 |
| Ethyl Acetate | 0.81 | 2.27 | 0.86 |
| 2-n-Butyl furan | -0.92 | 0.94 | 0.89 |
| 2-Pentyl-furan | 2.36 | 0.54 | -0.58 |
| Dodecane | 2.36 | 0.54 | -0.58 |
| Camphene | 1.34 | -2.24 | 0.99 |
| β-Terpinene | -1.43 | -0.31 | -0.23 |
| 3-Carene | -1.89 | -0.01 | 0.41 |
| o-Cymene | 2.34 | 0.89 | 0.57 |
| 2,4-Dimethyl-1-decene | 0.81 | 2.27 | 0.86 |
| Dimethyl ether | 0.34 | 1.96 | 0.30 |
| 4-Methyl-2-propylphenol | 0.22 | 0.94 | 1.71 |
| α-Limonene di-epoxide | -0.22 | -0.43 | -1.43 |
| Unknown2 | -1.11 | -0.33 | 0.24 |
| Unknown4 | -0.92 | 0.94 | 0.89 |
| Unknown6 | -1.15 | -1.91 | -0.98 |
| Unknown7 | 0.34 | 1.96 | 0.30 |
| Growth | 0.45 | 1.88 | -0.14 |

**Table S7.** Identified volatile compounds in unfermented and fermented plant-based milks, aroma attributes, odor groups, and the odor threshold in air.

| **Nr** | **Compound name*** | **RI, cal (min)** | **Aroma description** | **Odour group** | **Odour threshold (ppbv)** | **Identification** |
| --- | --- | --- | --- | --- | --- | --- |
| **Aldehydes** | |  |  |  |  |  |
| 1 | 3-Methyl-butanal | 648 | Malty [[9](#_ENREF_9)], fatty [[10](#_ENREF_10)], cocoa [[11](#_ENREF_11)], fruity [[11](#_ENREF_11)] | 1 | 11 [[12](#_ENREF_12)] | MS, RI |
| 2 | 2-Methyl-butanal | 654 | Malty [[9](#_ENREF_9)], cocoa [[11](#_ENREF_11)] | 1 | 11 [[12](#_ENREF_12)] | MS, RI |
| 3 | Hexanal | 799 | Green [[3](#_ENREF_3), [9](#_ENREF_9), [15](#_ENREF_15), [16](#_ENREF_16)], grassy [[3](#_ENREF_3), [9](#_ENREF_9), [14](#_ENREF_14), [17](#_ENREF_17)], nutty [[18](#_ENREF_18)], fat [[17](#_ENREF_17)], oxidized oil [[19](#_ENREF_19)] | 6 | 0.28 [[20](#_ENREF_20)] | MS, STD, RI |
| 4 | 2,4-Heptadienal^3^ | 1010 | Fatty [[11](#_ENREF_11)], creamy [[11](#_ENREF_11)], green [[11](#_ENREF_11)] | 3 | 8 [[12](#_ENREF_12)] | MS, RI |
| 5 | 2-Heptenal | 957 | Green [[11](#_ENREF_11), [21](#_ENREF_21)], fatty [[21](#_ENREF_21)] | 6 | 19 [[12](#_ENREF_12)] | MS, RI |
| 6 | Heptanal | 904 | Green [[11](#_ENREF_11)], citrus [[17](#_ENREF_17)], fatty [[17](#_ENREF_17)], floral [[15](#_ENREF_15)], rancid [[14](#_ENREF_14), [17](#_ENREF_17)] | 6 | 0.18 [[20](#_ENREF_20)] | MS, STD, RI |
| 7 | Benzaldehyde | 959 | Sweet [[3](#_ENREF_3), [9](#_ENREF_9)], fruity [[11](#_ENREF_11)], almond [[3](#_ENREF_3), [9](#_ENREF_9)] | 1 | 20 [[15](#_ENREF_15)] | MS, STD, RI |
| 8 | 2-Octenal | 1058 | Fatty [[11](#_ENREF_11)] | 3 | 0.53 [[15](#_ENREF_15)] | MS, RI |
| 9 | Phenylacetaldehyde | 1043 | Floral [[9](#_ENREF_9)], sweet [[3](#_ENREF_3), [9](#_ENREF_9)], honey [[9](#_ENREF_9)] | 2 | 4 [[20](#_ENREF_20)] | MS, STD, RI |
| 10 | 4-Ethyl-benzaldehyde | 1161 | Fruity [[11](#_ENREF_11)] | 1 | 13 [[20](#_ENREF_20)] | MS, RI |
| 11 | 2-Nonenal | 1158 | Green [[11](#_ENREF_11), [22](#_ENREF_22)], musty [[22](#_ENREF_22)], fatty [[11](#_ENREF_11)], | 6 | 0.02 [[15](#_ENREF_15)] | MS, RI |
| 12 | Nonanal | 1103 | Citrus [[14](#_ENREF_14), [23](#_ENREF_23)], floral [[10](#_ENREF_10), [14](#_ENREF_14), [22](#_ENREF_22), [23](#_ENREF_23)], fatty [[15](#_ENREF_15)], green [[23](#_ENREF_23)], smoky [[13](#_ENREF_13)] | 1 | 0.34 [[20](#_ENREF_20)] | MS, STD, RI |
| 13 | 2,4-Decadienal^4^ | 1312 | Grassy [[3](#_ENREF_3)], fatty [[11](#_ENREF_11)], melon [[3](#_ENREF_3)], aldehyde [[3](#_ENREF_3)] | 6 | 0.07 [[20](#_ENREF_20)] | MS, RI |
| 14 | Decanal | 1202 | Fatty [[9](#_ENREF_9)], floral [[10](#_ENREF_10)] | 3 | 0.10 [[20](#_ENREF_20)] | MS, RI |
| **Alcohols** | |  |  |  |  |  |
| 1 | [S, S]-2,3-Butanediol^1,2^ | 780 | Creamy [[11](#_ENREF_11), [24](#_ENREF_24)], buttery [[11](#_ENREF_11), [24](#_ENREF_24)] | 3 | 49 [[24](#_ENREF_24)] | MS, RI |
| 2 | 3-Methyl-2-buten-1-ol ^2^ | 773 | Fruity [[11](#_ENREF_11)] | 1 | 173 [[10](#_ENREF_10)] | MS, RI |
| 3 | 3-Methyl-1-butanol ^2,4^ | 733 | Fruity [[3](#_ENREF_3), [25](#_ENREF_25)], banana [[3](#_ENREF_3)], whiskey [[3](#_ENREF_3), [10](#_ENREF_10)], floral [[9](#_ENREF_9), [25](#_ENREF_25)], fermented [[11](#_ENREF_11)] | 1 | 68 [[26](#_ENREF_26)] | MS, RI |
| 4 | 2-Methyl-1-butanol ^1,4^ | 734 | Ethereal [[11](#_ENREF_11)], floral [[25](#_ENREF_25)] | 1 | 68 [[26](#_ENREF_26)] | MS, RI |
| 5 | 1-Pentanol | 765 | Fermented [[11](#_ENREF_11)], sweet [[10](#_ENREF_10)], fruity [[23](#_ENREF_23)], balsamic [[23](#_ENREF_23)], alcoholic [[14](#_ENREF_14)] | 1 | 43 [[15](#_ENREF_15)] | MS, STD, RI |
| 6 | 1-Hexanol | 869 | Fruity [[11](#_ENREF_11), [23](#_ENREF_23), [25](#_ENREF_25)] lemon [[3](#_ENREF_3), [9](#_ENREF_9)] | 1 | 6 [[27](#_ENREF_27)] | MS, STD, RI |
| 7 | 2-Heptanol^1,3,4^ | 900 | Citrus [[11](#_ENREF_11)], fruity [[24](#_ENREF_24), [28](#_ENREF_28)], herbal [[28](#_ENREF_28)] | 1 | 41 [[20](#_ENREF_20)] | MS, RI |
| 8 | 1-Heptanol | 970 | Light green [[3](#_ENREF_3)], mushroom [[14](#_ENREF_14)], rancid [[3](#_ENREF_3)] | 6 | 3 [[20](#_ENREF_20)] | MS, STD, RI |
| 9 | Benzyl alcohol^2,3^ | 1033 | Floral [[25](#_ENREF_25)] | 2 | 1 × 10^4^ [[20](#_ENREF_20)] | MS, RI |
| 10 | 2-Methyl-3-hexanol^1^ | 859 | Unknown | 9 |  | MS, RI |
| 11 | Phenylethyl alcohol^2,4^ | 1112 | Floral [[3](#_ENREF_3), [11](#_ENREF_11)], rose [[3](#_ENREF_3)], honey [[3](#_ENREF_3)] | 2 | 0.02 [[20](#_ENREF_20)] | MS, STD, RI |
| 12 | 1-Octen-3-ol | 979 | Earthy [[11](#_ENREF_11)], mushroom [[3](#_ENREF_3), [9](#_ENREF_9)] | 7 | 0.52 [[15](#_ENREF_15)] | MS, RI |
| 13 | 2-Octen-1-ol^1,2,3,4^ | 1067 | Cucumber [[3](#_ENREF_3)] | 2 | 40 [[20](#_ENREF_20)] | MS, RI |
| 14 | 3-Octanol^4^ | 996 | Earthy [[11](#_ENREF_11)] | 7 | 27 [[27](#_ENREF_27)] | MS, RI |
| 15 | 2-Ethyl-1-hexanol^4^ | 1028 | Citrus [[11](#_ENREF_11)], floral [[28](#_ENREF_28)] | 1 | 130 | MS, RI |
| 16 | 1-Octanol | 1070 | Waxy [[11](#_ENREF_11)], aldehyde [[11](#_ENREF_11)], fruity [[16](#_ENREF_16)], floral [[16](#_ENREF_16)] | 3 | 2.7 [[20](#_ENREF_20)] | MS, RI |
| 17 | 1-Nonanol^2,4^ | 1171 | Citrus, rose [[3](#_ENREF_3)] | 1 | 0.9 [[27](#_ENREF_27)] | MS, RI |
| 18 | Eugenol^4^ | 1368 | Pleasant spicy [[3](#_ENREF_3), [11](#_ENREF_11)], clove-like [[3](#_ENREF_3), [11](#_ENREF_11)] | 2 | 6 [[20](#_ENREF_20)] | MS, STD, RI |
| 19 | Cherry-propanol | 1183 | Fruity [[11](#_ENREF_11)] | 1 |  | MS, RI |
| 20 | Trans-pinocarveol | 1138 | Woody [[11](#_ENREF_11)], balsamic [[11](#_ENREF_11)] | 5 |  | MS, RI |
| 21 | *cis*-Verbenol | 1166 | Balsamic [[11](#_ENREF_11)], pine [[11](#_ENREF_11)] | 5 |  | MS, RI |
| 22 | (-)-Myrtenol | 1195 | Woody [[11](#_ENREF_11)], minty [[29](#_ENREF_29)], camphoraceous [[29](#_ENREF_29)] | 5 | 0.32 [[28](#_ENREF_28)] | MS, RI |
| 23 | Myrtenol | 1203 | Woody [[11](#_ENREF_11)], herbal [[11](#_ENREF_11)], floral [[28](#_ENREF_28)] | 5 | 0.32 [[28](#_ENREF_28)] | MS, RI |
| 24 | Linalool | 1100 | Floral [[11](#_ENREF_11), [25](#_ENREF_25), [28](#_ENREF_28), [30](#_ENREF_30)], fruity [[11](#_ENREF_11), [25](#_ENREF_25), [28](#_ENREF_28)] | 2 | 4 [[20](#_ENREF_20)] | MS, RI |
| 25 | Terpinen-4-ol | 1176 | Balsamic [[25](#_ENREF_25)], woody [[25](#_ENREF_25), [30](#_ENREF_30)], green [[25](#_ENREF_25)], fatty [[25](#_ENREF_25)], fruity [[30](#_ENREF_30)], floral [[30](#_ENREF_30)], spicy [[11](#_ENREF_11)] | 5 | 150 [[31](#_ENREF_31)] | MS, RI |
| 26 | 3-Ethyl-4-nonanol^2,3^ | 1094 | Unknown | 9 |  | MS, RI |
| **Ketones** | |  |  |  |  |  |
| 1 | Methyl-vinyl-ketone | 734 | Pungent [[11](#_ENREF_11)], sweet [[11](#_ENREF_11)] | 8 |  | MS |
| 2 | 2,3-Butanedione^2,3,4^ | 440 | Buttery [[11](#_ENREF_11), [18](#_ENREF_18)], sweet [[11](#_ENREF_11)], creamy [[11](#_ENREF_11)] | 3 | 0.3 [[20](#_ENREF_20)] | MS, RI |
| 3 | 3-Hydroxybutan-2-one^2,4^ | 706 | Buttery [[3](#_ENREF_3), [11](#_ENREF_11)], creamy [[3](#_ENREF_3), [10](#_ENREF_10), [11](#_ENREF_11)], sweet [[17](#_ENREF_17)], toasted [[17](#_ENREF_17)] | 3 | 0.3 [[20](#_ENREF_20)] | MS, RI |
| 4 | 2,3-Pentanedione^3^ | 694 | Buttery [[11](#_ENREF_11), [18](#_ENREF_18)], sweet [[18](#_ENREF_18)] | 3 | 12 [[10](#_ENREF_10)] | MS, RI |
| 5 | 2-Hexanone^1, 2^ | 800 | Fruity [[11](#_ENREF_11)] | 1 | 24 [[20](#_ENREF_20)] | MS, RI |
| 6 | 2,3-Heptanedione^1^ | 836 | Buttery [[11](#_ENREF_11)] | 3 |  | MS, RI |
| 7 | 3,6-Heptanedione^2^ | 1062 | Buttery [[11](#_ENREF_11)] | 3 |  | MS, RI |
| 8 | 2-Heptanone | 891 | Fruity [[9](#_ENREF_9), [15](#_ENREF_15)], floral [[18](#_ENREF_18)], sweet [[15](#_ENREF_15)], cheesy [[11](#_ENREF_11)] | 1 | 0.76 [[32](#_ENREF_32)] | MS, RI |
| 9 | 1-(2-Furanyl)-1-propanone^2^ | 1031 | Fruity [[11](#_ENREF_11)] | 1 |  | MS, RI |
| 10 | 3,5-Octadien-2-one | 1092 | Fruity [[11](#_ENREF_11)], fatty [[11](#_ENREF_11)], green [[3](#_ENREF_3), [11](#_ENREF_11)], earthy [[3](#_ENREF_3)] | 1 | 5 [[20](#_ENREF_20)] | MS, RI |
| 11 | 6-Methyl-5-hepten-2-one^3^ | 987 | Citrus [[11](#_ENREF_11), [21](#_ENREF_21)], musty [[10](#_ENREF_10), [21](#_ENREF_21)], green [[21](#_ENREF_21)] | 1 | 1 [[12](#_ENREF_12)] | MS, RI |
| 12 | 5-Methyl-3-hepten-2-one | 1039 | Unknown | 9 |  | MS, RI |
| 13 | 3-Octanone^4^ | 987 | Earthy [[9](#_ENREF_9)], mushroom-like [[11](#_ENREF_11)], fresh [[11](#_ENREF_11)], herbal [[11](#_ENREF_11)], ripe banana [[11](#_ENREF_11)] | 7 | 24 [[20](#_ENREF_20)] | MS, RI |
| 14 | Isoacetovanillone | 1158 | Unknown | 9 |  | MS, RI |
| 15 | 2-Nonanone^4^ | 1091 | Sweet [[10](#_ENREF_10), [21](#_ENREF_21)], fruity [[15](#_ENREF_15), [21](#_ENREF_21)], floral [[15](#_ENREF_15)], green [[17](#_ENREF_17), [21](#_ENREF_21)], hot milk [[17](#_ENREF_17)], soap [[17](#_ENREF_17)] | 1 | 5 [[20](#_ENREF_20)] | MS, RI |
| 16 | 5,6-Dehydrocamphor | 1095 | Unknown | 9 |  | MS, RI |
| 17 | Pinocarvone | 1162 | Camphoraceous [[33](#_ENREF_33)], fresh [[33](#_ENREF_33)] | 5 |  | MS, RI |
| 18 | D-Verbenone | 1208 | Camphoraceous [[11](#_ENREF_11)], minty [[11](#_ENREF_11), [33](#_ENREF_33)], spicy [[11](#_ENREF_11), [33](#_ENREF_33)] | 5 |  | MS, RI |
| **Organic acids** | |  |  |  |  |  |
| 1 | Acetic acid^1,2,3,4^ | 401 | Acidic [[9](#_ENREF_9), [11](#_ENREF_11)], sour [[11](#_ENREF_11)], vinegar [[10](#_ENREF_10), [11](#_ENREF_11), [34](#_ENREF_34)] | 4 | 6 [[27](#_ENREF_27)] | MS, STD, RI |
| 2 | 3-Methyl-butanoic acid^1,3^ | 843 | Cheesy [[9](#_ENREF_9), [34](#_ENREF_34)] | 4 | 0.08 [[27](#_ENREF_27)] | MS, RI |
| 3 | 2-Methyl-butanoic acid^2,3^ | 853 | Sour [[11](#_ENREF_11)], cheesy [[11](#_ENREF_11)], fermented [[11](#_ENREF_11)] | 4 | 0.04 [[27](#_ENREF_27)] | MS, RI |
| 4 | Pentanoic acid^1^ | 882 | Cheesy [[11](#_ENREF_11)], acidic [[11](#_ENREF_11)], unpleasant [[10](#_ENREF_10)] | 4 | 8.90 [[26](#_ENREF_26)] | MS, RI |
| 5 | Hexanoic acid^2,3^ | 982 | Cheesy [[11](#_ENREF_11), [24](#_ENREF_24), [28](#_ENREF_28)], fatty [[11](#_ENREF_11), [24](#_ENREF_24), [28](#_ENREF_28)], sour [[3](#_ENREF_3)], sharp [[3](#_ENREF_3)], rancid [[3](#_ENREF_3), [28](#_ENREF_28)] | 4 | 0.60 [[27](#_ENREF_27)] | MS, RI |
| 6 | Octanoic acid^2,3^ | 1167 | Cheesy [[10](#_ENREF_10), [24](#_ENREF_24)], fatty [[11](#_ENREF_11), [24](#_ENREF_24)], sweaty [[3](#_ENREF_3)] | 4 | 27 [[24](#_ENREF_24)] | MS, RI |
| 7 | Nonanoic acid | 1269 | Waxy [[11](#_ENREF_11)], earthy [[10](#_ENREF_10)] | 3 | 1.9 [[32](#_ENREF_32)] | MS, RI |
| **Esters** | |  |  |  |  |  |
| 1 | Ethylacetate | 916 | Sweet [[11](#_ENREF_11), [21](#_ENREF_21)], fruity [[11](#_ENREF_11), [21](#_ENREF_21)], mild [[10](#_ENREF_10)] | 1 | 870 [[27](#_ENREF_27)] | MS, RI |
| 2 | Ethyllactate^1^ | 791 | Fruity [[11](#_ENREF_11)], buttery [[11](#_ENREF_11)] | 1 | 5 × 10^4^ [[20](#_ENREF_20)] | MS, RI |
| 3 | Ethyl 2-methylbutyrate | 850 | Fruity [[11](#_ENREF_11)] | 1 | 0.01 [[20](#_ENREF_20)] | MS, RI |
| 4 | Hexyl acetate | 1014 | Fruity [[11](#_ENREF_11), [21](#_ENREF_21)], sweet [[21](#_ENREF_21)] | 1 | 1.8 [[27](#_ENREF_27)] | MS, RI |
| 5 | Verbenylacetate | 1144 | Unknown | 9 |  | MS, RI |
| 6 | Bornyl acetate^2^ | 1201 | Balsamic [[11](#_ENREF_11)], camphoraceous [[11](#_ENREF_11)] | 5 | 75 [[20](#_ENREF_20)] | MS |
| 7 | Epoxy-alpha-terpenylacetate | 1130 | Unknown | 9 |  | MS, RI |
| **Furans** | |  |  |  |  |  |
| 1 | 2-Ethyl-furan^3^ | 699 | Chemical [[11](#_ENREF_11)], sweet [[35](#_ENREF_35)], coffee-like [[35](#_ENREF_35)] | 8 | 1.3 × 10^6^ [[35](#_ENREF_35)] | MS, RI |
| 2 | 2-Ethyl-5-methylfuran^2^ | 773 | Grassy [[11](#_ENREF_11)] | 8 |  | MS, RI |
| 3 | 2-Acetyl-5-methylfuran | 855 | Nutty [[11](#_ENREF_11)] | 5 |  | MS, RI |
| 4 | 2-n-Butyl furan^1^ | 892 | Spicy [[11](#_ENREF_11)], fruity [[11](#_ENREF_11)], wine-like [[11](#_ENREF_11)] | 8 | 1 × 10^5^ [[35](#_ENREF_35)] | MS, RI |
| 5 | 2-(1-Pentenyl)-furan^3^ | 1001 | Roasted [[11](#_ENREF_11)] | 5 |  | MS, RI |
| 6 | 2-Pentyl-furan | 991 | Beany [[16](#_ENREF_16)], green [[3](#_ENREF_3), [24](#_ENREF_24)], grassy [[19](#_ENREF_19)], nutty [[16](#_ENREF_16)], fatty [[3](#_ENREF_3)] | 6 | 3.4 [[15](#_ENREF_15)] | MS, RI |
| 7 | 2-n-Heptylfuran | 1190 | Green [[11](#_ENREF_11)], fatty [[11](#_ENREF_11)] | 6 |  | MS, RI |
| **Alkanes** | |  |  |  |  |  |
| 1 | Undecane | 1098 | Unknown | 9 | 620 [[20](#_ENREF_20)] | MS, STD, RI |
| 2 | Dodecane | 1196 | Unknown | 9 | 110 [[27](#_ENREF_27)] | MS, STD, RI |
| 3 | Tridecane | 1295 | Unknown | 9 |  | MS, STD, RI |
| **Alkenes** | |  |  |  |  |  |
| 1 | a -Pinene | 933 | Pine [[13](#_ENREF_13), [17](#_ENREF_17), [29](#_ENREF_29), [36](#_ENREF_36)], woody [[11](#_ENREF_11)], herbal [[11](#_ENREF_11)], fresh [[11](#_ENREF_11)], fruity [[36](#_ENREF_36), [37](#_ENREF_37)], | 5 | 18 [[27](#_ENREF_27)] | MS, STD, RI |
| 2 | Camphene | 950 | Woody [[1,](#_ENREF_11) [20](#_ENREF_20)], herbal [[11](#_ENREF_11)], camphoraceous [[11](#_ENREF_11), [36](#_ENREF_36), [37](#_ENREF_37), [38](#_ENREF_38)] | 5 | 130 [[12](#_ENREF_12)] | MS, RI |
| 3 | β-Terpinene | 975 | Terpenic [[38](#_ENREF_38)], fatty [[38](#_ENREF_38)] | 5 | 130 [[12](#_ENREF_12)] | MS, RI |
| 4 | 3-Carene | 1009 | Citrus [[11](#_ENREF_11)], sweet [[33](#_ENREF_33)] | 1 | 140 [[12](#_ENREF_12)] | MS, RI |
| 5 | p-Cymene | 1024 | Fruity [[36](#_ENREF_36)], fresh [[11](#_ENREF_11)], citrus [[11](#_ENREF_11), [30](#_ENREF_30)], terpenic [[11](#_ENREF_11)], floral [[38](#_ENREF_38)], fragrant [[38](#_ENREF_38)] | 1 | 57 [[20](#_ENREF_20)] | MS, RI |
| 6 | D-Limonene | 1028 | Citrus [[10](#_ENREF_10), [19](#_ENREF_19), [30](#_ENREF_30)], fresh [[11](#_ENREF_11)], sweet [[11](#_ENREF_11)] | 1 | 38 [[27](#_ENREF_27)] | MS, STD, RI |
| 7 | γ-Terpinene | 1058 | Terpenic [[11](#_ENREF_11), [30](#_ENREF_30)], citrus [[30](#_ENREF_30)], herbal [[30](#_ENREF_30)] | 5 | 5 × 10^4^ [[31](#_ENREF_31)] | MS, RI |
| 8 | α-Thujene^2^ | 1121 | Woody [[11](#_ENREF_11)], herbal [[30](#_ENREF_30), [33](#_ENREF_33)], green [[30](#_ENREF_30), [33](#_ENREF_33)] | 5 |  | MS, RI |
| 9 | Alloocimene^1^ | 1128 | Floral [[11](#_ENREF_11)], sweet [[11](#_ENREF_11)], nut [[11](#_ENREF_11)] | 2 | 1.8 × 10^4^ [[39](#_ENREF_39)] | MS, RI |
| 10 | β-Thujene | 953 | Unknown |  |  | MS, RI |
| 11 | 2,4-Dimethyl-1-decene | 1083 | Unknown | 9 |  | MS, RI |
| 12 | β-Gurjunene | 1434 | Unknown | 9 |  | MS, RI |
| **Others** | |  |  |  |  |  |
| 1 | Dimethyl ether^2,3,4^ | 478 | Ethereal [[11](#_ENREF_11)] | 1 | 5 × 10^5^ [[20](#_ENREF_20)] | MS, RI |
| 2 | 4-Ethenyl-1,2-dimethyl-benzene | 1088 | Unknown | 9 |  | MS, RI |
| 3 | 4-Methyl-2-propylphenol^1^ | 1314 | Unknown | 9 |  | MS, RI |
| 4 | α-Limonene-di-epoxide | 1031 | Citrus [[11](#_ENREF_11)] | 1 |  | MS, RI |
| 5 | Unknown^3^ | 735 |  |  |  |  |
| 6 | Unknown | 989 |  |  |  |  |
| 7 | Unknown ^4^ | 1028 |  |  |  |  |
| 8 | Unknown | 1039 |  |  |  |  |
| 9 | Unknown | 1079 |  |  |  |  |
| 10 | Unknown | 1085 |  |  |  |  |
| 11 | Unknown | 1332 |  |  |  |  |
| 12 | Unknown | 1341 |  |  |  |  |

* The superscripted number represents newly formed volatiles during fermentation of oat milk (1), sunflower seed milk (2), pea milk (3), and faba milk (4).

** Odor groups: 1) fruity, citrus, sweet, malty, ethereal; 2) floral, nice green; 3) buttery, fatty, waxy, creamy; 4) cheesy, sour; 5) nutty, woody, minty, toasted, turpentine, balsamic, camphoraceous; 6) green, grassy, bean-like, herbal; 7) earthy, mushroom; 8) pungent, spicy, sharp, phenolic; and 9) unknown odour.

**Table S8.** Calculation of the odor threshold (OT) of the volatiles in air (ppbv).

|  | OT  1 | OT  2 | OT  3 | OT  4 | OT  5 | OT  6 | OT  7 | OT  8 | OT  9 | OT  10 | OT  11 | OT  12 | OT  13 | OT  14 | Used |
| --- | --- | --- | --- | --- | --- | --- | --- | --- | --- | --- | --- | --- | --- | --- | --- |
| 3-Methyl-butanal |  | 0.1 | 11.0 |  |  |  |  |  |  |  |  |  |  |  | 11.0 |
| 2-Methyl-butanal |  |  | 11.0 |  |  |  |  |  |  |  |  |  |  |  | 11.0 |
| Hexanal |  | 0.3 |  |  |  | 9.7 |  |  |  |  |  |  | 12.6 | 0.3 | 0.3 |
| 2,4-Heptadienal |  | . | 8.0 |  |  |  |  |  |  |  |  |  | 8.5 |  | 8.0 |
| 2-Heptenal |  | . | 19.0 |  |  |  |  |  |  |  |  |  | 19.4 |  | 19.4 |
| Heptanal |  | 0.2 | 0.2 |  |  |  |  |  |  |  |  |  | 10.0 | 0.8 | 0.2 |
| Benzaldehyde |  | 100.0 |  |  |  |  |  |  |  |  |  |  | 43.4 | 19.8 | 19.8 |
| 2-Octenal |  | . | 1.0 |  |  |  |  |  |  |  |  |  |  | 0.5 | 0.5 |
| Phenylacetaldehyde |  | 4.0 |  |  |  |  |  |  |  |  |  |  |  |  | 4.0 |
| 4-Ethyl-benzaldehyde |  | 13.0 |  |  |  |  |  |  |  |  |  |  |  |  | 13.0 |
| 2-Nonenal |  | 0.1 | 0.0 |  |  |  |  |  |  |  |  |  |  | 0.0 | 0.0 |
| Nonanal |  | 0.3 | 0.5 |  |  |  |  |  |  |  |  |  | 2.1 | 0.5 | 0.3 |
| 2,4-Decadienal |  | 0.1 | 0.4 |  |  |  |  |  |  |  |  |  |  | 0.4 | 0.1 |
| Decanal |  | 0. | 0.4 |  |  |  |  |  |  |  |  |  | 0.7 |  | 0.1 |
| [S,S]-2,3-Butanediol |  | . |  |  | 48.9 |  |  |  |  |  |  |  |  |  | 48.9 |
| 3-Methyl-2-buten-1-ol |  | . |  |  |  |  |  | 172.5 |  |  |  |  |  |  | 172.5 |
| 3-Methyl-1-butanol |  |  |  |  |  | 67.8 |  |  |  |  |  |  |  |  | 67.8 |
| 2-Methyl-1-butanol |  |  |  |  |  | 67.8 |  |  |  |  |  |  |  |  | 67.8 |
| 1-Pentanol |  | 100.0 |  |  | 9672.8 | 604.6 |  |  |  |  |  |  | 1008.8 | 43.0 | 43.0 |
| 1-Hexanol | 6.0 | 6.0 |  |  | 188.7 | 429.0 |  |  |  |  |  |  | 87.3 |  | 6.0 |
| 2-Heptanol |  | 41.0 |  |  |  |  |  |  |  |  |  |  |  |  | 41.0 |
| 1-Heptanol |  | 3.0 |  |  |  |  |  |  |  |  |  |  |  |  | 3.0 |
| Benzyl alcohol |  | 0.0 |  |  | 298063.2 | 14903.2 |  |  |  |  |  |  |  |  | 10000.0 |
| 2-Methyl-3-hexanol |  |  |  |  |  |  |  |  |  |  |  |  |  |  |  |
| Phenylethyl alcohol |  |  |  |  |  |  | 173.5 |  |  |  |  |  |  |  | 173.0 |
| 1-Octen-3-ol |  | 14.0 |  |  |  |  |  |  |  |  |  |  |  | 0.5 | 0.5 |
| 2-Octen-1-ol |  | 40.0 |  |  |  |  |  |  |  |  |  |  |  |  | 40.0 |
| 3-Octanol | 27.0 |  |  |  |  |  |  |  |  |  |  |  |  |  | 27.0 |
| 2-Ethyl-1-hexanol |  | 130.0 |  |  |  |  |  |  |  |  |  |  |  |  | 130.0 |
| 1-Octanol | 27.0 | 2.7 |  |  |  |  |  |  |  |  |  |  | 13.9 | 4.2 | 2.7 |
| 1-Nonanol | 0.9 | 0.9 |  |  |  |  |  |  |  |  |  |  |  | 3.1 | 0.9 |
| Eugenol |  | 6.0 |  |  |  |  |  |  |  |  |  |  |  |  | 6.0 |
| Cherry propanol |  | . |  |  |  |  |  |  |  |  |  |  |  |  |  |
| laevo-Pinocarveol |  |  |  |  |  |  |  |  |  |  |  |  |  |  |  |
| cis-Verbenol |  |  |  |  |  |  |  |  |  |  |  |  |  |  |  |
| (-)-Myrtenol |  |  |  |  |  |  |  |  |  |  |  | 0.3 |  |  | 7.0 |
| Myrtenol |  |  |  |  |  |  |  |  |  |  |  | 0.3 |  |  | 7.0 |
| Linalool |  | 4.0 |  |  | 6.5 |  |  |  |  |  |  |  |  |  | 4.0 |
| Terpinen-4-ol |  |  |  |  |  |  |  |  | 150.3 |  |  |  |  |  | 150.3 |
| 3-Ethyl-4-nonanol |  |  |  |  |  |  |  |  |  |  |  |  |  |  |  |
| Methyl vinyl ketone |  |  |  |  |  |  |  |  |  |  |  |  |  |  |  |
| 2,3-Butanedione |  | 0.3 |  |  |  |  |  | 0.0 |  |  |  |  |  |  | 0.3 |
| 3-Hydroxy-2-butanone |  |  |  |  | 17182.6 | 91.6 |  |  |  |  | 1.6 |  |  |  | 1.6 |
| 2,3-Pentanedione |  | 20.0 |  |  |  |  |  | 11.6 |  |  |  |  |  |  | 11.6 |
| 2-Hexanone |  | 24-80 |  |  |  |  |  |  |  |  |  |  |  |  | 24.0 |
| 2,3-Heptanedione |  |  |  |  |  |  |  |  |  |  |  |  |  |  |  |
| 3,6-Heptanedione |  |  |  |  |  |  |  |  |  |  |  |  |  |  |  |
| 2-Heptanone |  | 1.0 | 1.0 |  |  |  |  |  |  |  |  |  | 0.8 |  | 0.8 |
| 1-(2-Furanyl)-1-propanone |  |  |  |  |  |  |  |  |  |  |  |  |  |  |  |
| 3,5-Octadien-2-one |  | 5.1 |  |  |  |  |  |  |  |  |  |  |  |  | 5.1 |
| 6-Methyl-5-hepten-2-one |  | 50.0 | 1.0 |  |  |  |  |  |  |  |  |  |  |  | 1.0 |
| 5-Methyl-3-hepten-2-one |  |  |  |  |  |  |  |  |  |  |  |  |  |  |  |
| 3-Octanone |  | 21-50 |  |  |  |  |  |  |  |  |  |  |  |  | 21.0 |
| Isoacetovanillone |  |  |  |  |  |  |  |  |  |  |  |  |  |  |  |
| 2-Nonanone4 |  | 5.0 |  |  |  |  |  |  |  |  |  |  | 5.4 |  | 5.0 |
| 5,6-Dehydrocamphor |  |  |  |  |  |  |  |  |  |  |  |  |  |  |  |
| Pinocarvone |  |  |  |  |  |  |  |  |  |  |  |  |  |  |  |
| D-Verbenone |  |  |  |  |  |  |  |  |  |  |  |  |  |  |  |
| Acetic acid | 6.0 | 6.0 | 162.0 | 480.0 |  |  |  |  |  |  |  |  | 570.9 |  | 6.0 |
| 3-Methyl-butanoic acid | 0.1 | 160.0 |  | 19.0 | 183.4 |  |  |  |  |  |  |  |  |  | 0.1 |
| 2-Methyl-butanoic acid | 0.0 | 10.0 |  |  |  |  |  |  |  |  |  |  |  |  | 0.0 |
| Pentanoic acid |  | 0.0 | 33.0 | 9.0 |  | 8.9 |  |  |  |  |  |  |  |  | 0.0 |
| Hexanoic acid | 0.6 | 0.6 |  |  | 15.1 |  |  |  |  |  |  |  | 639.8 |  | 0.6 |
| Octanoic Acid |  | 910.0 |  |  | 26.6 |  |  |  |  |  |  |  |  |  | 26.6 |
| Nonanoic acid |  | 3.0 |  |  |  |  |  |  |  |  |  |  | 1.9 |  | 1.9 |
| Ethyl Acetate | 870 | 870 | 1000 | 3900 |  | 3844 | 14416 |  |  |  |  |  |  |  | 870 |
| Ethyl lactate |  | 50000.0 |  |  |  |  |  |  |  |  |  |  |  |  | 50000.0 |
| Ethyl 2-methylbutyrate |  | 0.0 |  |  |  |  |  | 0.5 |  |  |  |  |  |  | 0.0 |
| Hexyl acetate | 1.8 |  | 307 |  | 8224 | 11.0 |  |  |  |  |  |  |  |  | 1.8 |
| Verbenyl acetate |  |  |  |  |  |  |  |  |  |  |  |  |  |  |  |
| Bornyl acetate |  | 75 |  |  |  |  |  |  |  |  |  |  |  |  | 75 |
| Epoxy-.alpha.-terpenyl acetate |  |  |  |  |  |  |  |  |  |  |  |  |  |  |  |
| 2-Ethyl-furan |  |  |  |  |  |  |  |  |  | 1265684 |  |  |  |  | 1265684 |
| 2-Ethyl-5-methylfuran |  |  |  |  |  |  |  |  |  |  |  |  |  |  |  |
| 2-Acetyl-5-methylfuran |  |  |  |  |  |  |  |  |  |  |  |  |  |  |  |
| 2-n-Butyl furan |  |  |  |  |  |  |  |  |  | 103723.6 |  |  |  |  | 103723.6 |
| 2-(1-Pentenyl)-furan |  |  |  |  |  |  |  |  |  |  |  |  |  |  |  |
| 2-Pentyl-furan |  | 6.0 |  |  |  |  |  |  |  | 178 |  |  | 32.4 | 3.4 | 3.4 |
| 2-n-Heptylfuran |  |  |  |  |  |  |  |  |  |  |  |  |  |  |  |
| Undecane | 870 | 620 |  |  |  |  |  |  |  |  |  |  |  |  | 620 |
| Dodecane | 110 | 110 |  |  |  |  |  |  |  |  |  |  | 770 |  | 110 |
| Tridecane |  |  |  |  |  |  |  |  |  |  |  |  |  |  |  |
| a-Pinene | 18 |  | 130 |  |  |  |  |  | 48051.7 |  |  |  |  |  | 18 |
| Camphene |  | 880 | 130 |  |  |  |  |  |  |  |  |  |  |  | 130 |
| β-Terpinene |  |  | 130 |  |  |  |  |  |  |  |  |  |  |  | 130 |
| 3-Carene |  |  | 140 |  |  |  |  |  |  |  |  |  |  |  | 140 |
| p-Cymene |  | 57 |  |  |  |  |  |  |  |  |  |  |  |  | 57 |
| D-Limonene | 38 | 38 | 130 |  |  |  |  |  |  |  |  |  | 130.6 |  | 38 |
| γ-Terpinene |  |  |  |  |  |  |  |  |  |  | 50185.2 |  |  |  | 50185.2 |
| α-Thujene |  |  |  |  |  |  |  |  |  |  |  |  |  |  |  |
| Alloocimene |  |  |  |  |  |  |  |  |  |  | 17786.7 |  |  |  | 17786.7 |
| β-Thujene |  |  |  |  |  |  |  |  |  |  |  |  |  |  |  |
| 2,4-Dimethyl-1-decene |  |  |  |  |  |  |  |  |  |  |  |  |  |  |  |
| β-Gurjunene |  |  |  |  |  |  |  |  |  |  |  |  |  |  |  |
| Methyl isocyanate |  |  |  |  |  |  |  |  |  |  |  |  |  |  |  |
| Dimethyl ether |  | 500000.0 |  |  |  |  |  |  |  |  |  |  |  |  | 500000.0 |
| 4-Ethenyl-1,2-dimethyl-benzene |  |  |  |  |  |  |  |  |  |  |  |  |  |  |  |
| 4-Methyl-2-propylphenol |  |  |  |  |  |  |  |  |  |  |  |  |  |  |  |
| α-Limonene diepoxide |  |  |  |  |  |  |  |  |  |  |  |  |  |  |  |
| 5-Methoxy-1,3-dimethyl-1H-pyrazole |  |  |  |  |  |  |  |  |  |  |  |  |  |  |  |
| Benzothiazole |  |  |  |  |  |  |  |  |  |  |  |  |  |  |  |
| 1,2-Benzisothiazole |  |  |  |  |  |  |  |  |  |  |  |  |  |  |  |
| Benzene, 1,3-bis(1,1-dimethylethyl)- |  |  |  |  |  |  |  |  |  |  |  |  |  |  |  |

OT1-14 were collected or calculated from following literature: OT1 [[9](#_ENREF_9)], OT2 [[10](#_ENREF_10)], OT3 [[11](#_ENREF_11)], OT4 [[12](#_ENREF_12)], OT5 [[13](#_ENREF_13)], OT6 [[14](#_ENREF_14)], OT7 [[15](#_ENREF_15)], OT8 [[16](#_ENREF_16)], OT9 [[17](#_ENREF_17)], OT10 [[18](#_ENREF_18)], OT11 [[19](#_ENREF_19)], OT12 [[20](#_ENREF_20)], OT13 [[21](#_ENREF_21)], OT14 [[22](#_ENREF_22)].

**Reference**

1. Jeleń HH, Majcher M, Dziadas M: **Microextraction techniques in the analysis of food flavor compounds: A review.** *Analytica chimica acta* 2012, **738:**13-26.

2. Merkle S, Kleeberg KK, Fritsche J: **Recent developments and applications of solid phase microextraction (SPME) in food and environmental analysis—a review.** *Chromatography* 2015, **2:**293-381.

3. Xu M, Jin Z, Lan Y, Rao J, Chen B: **HS-SPME-GC-MS/olfactometry combined with chemometrics to assess the impact of germination on flavor attributes of chickpea, lentil, and yellow pea flours.** *Food Chemistry* 2019, **280:**83-95.

4. Fischer E, Cachon R, Cayot N: **Effects of extraction pH on the volatile compounds from pea protein isolate: Semi-Quantification method using HS-SPME-GC-MS.** *Food Research International* 2021, **150:**110760.

5. Lee SM, Oh J, Hurh BS, Jeong GH, Shin YK, Kim YS: **Volatile compounds produced by *Lactobacillus paracasei* during oat fermentation.** *Journal of food science* 2016, **81:**C2915-C2922.

6. Wronkowska M, Rostek D, Lenkiewicz M, Kurantowicz E, Yaneva TG, Starowicz M: **Oat flour fermented by *Lactobacillus* strains–Kinetics of volatile compound formation and antioxidant capacity.** *Journal of Cereal Science* 2022, **103:**103392.

7. Tangyu M, Fritz M, Ye L, Aragão Börner R, Morin-Rivron D, Campos-Giménez E, Bolten CJ, Bogicevic B, Wittmann C: **Co-cultures of *Propionibacterium freudenreichii* and *Bacillus amyloliquefaciens* cooperatively upgrade sunflower seed milk to high levels of vitamin B_12_ and multiple co-benefits.** *Microbial cell factories* 2022, **21:**1-23.

8. Schindler S, Zelena K, Krings U, Bez J, Eisner P, Berger RG: **Improvement of the aroma of pea (*Pisum sativum*) protein extracts by lactic acid fermentation.** *Food Biotechnology* 2012, **26:**58-74.

9. Nagata Y, Takeuchi N: **Measurement of odor threshold by triangle odor bag method.** *Odor measurement review* 2003, **118:**118-127.

10. **Chemical Book** [<https://www.chemicalbook.com/>]

11. Yan J, Alewijn M, van Ruth SM: **From extra virgin olive oil to refined products: Intensity and balance shifts of the volatile compounds versus odor.** *Molecules* 2020, **25:**2469.

12. Amoore JE, Hautala E: **Odor as an ald to chemical safety: odor thresholds compared with threshold limit values and volatilities for 214 industrial chemicals in air and water dilution.** *Journal of applied toxicology* 1983, **3:**272-290.

13. Cai J, Zhu B-Q, Wang Y-H, Lu L, Lan Y-B, Reeves MJ, Duan C-Q: **Influence of pre-fermentation cold maceration treatment on aroma compounds of Cabernet Sauvignon wines fermented in different industrial scale fermenters.** *Food chemistry* 2014, **154:**217-229.

14. van Gemert LJ: *Odour thresholds: compilations of odour threshold values in air, water and other media.* Zeist, Netherlands: Oliemans Punter & Partners; 2011.

15. Ouyang X, Yuan G, Ren J, Wang L, Wang M, Li Y, Zhang B, Zhu B: **Aromatic compounds and organoleptic features of fermented wolfberry wine: Effects of maceration time.** *International Journal of Food Properties* 2017, **20:**2234-2248.

16. Liu C, Yang P, Wang H, Song H: **Identification of odor compounds and odor-active compounds of yogurt using DHS, SPME, SAFE, and SBSE/GC-O-MS.** *LWT-Food Science and Technology* 2022, **154:**112689.

17. Tamura H, Boonbumrung S, Yoshizawa T, Varanyanond W: **The volatile constituents in the peel and pulp of a green Thai mango, Khieo Sawoei cultivar (*Mangifera indica* L.).** *Food Science and Technology Research* 2001, **7:**72-77.

18. Maga JA, Katz I: **Furans in foods.** *Critical Reviews in Food Science and Nutrition* 1979, **11:**355-400.

19. Yang Y-N, Zheng F-P, Yu A-N, Sun B-G: **Changes of the free and bound volatile compounds in *Rubus corchorifolius* L. f. fruit during ripening.** *Food chemistry* 2019, **287:**232-240.

20. Wu Y, Duan S, Zhao L, Gao Z, Luo M, Song S, Xu W, Zhang C, Ma C, Wang S: **Aroma characterization based on aromatic series analysis in table grapes.** *Scientific reports* 2016, **6:**1-16.

21. Xu L, Yu X, Li M, Chen J, Wang X: **Monitoring oxidative stability and changes in key volatile compounds in edible oils during ambient storage through HS-SPME/GC–MS.** *International Journal of Food Properties* 2017, **20:**S2926-S2938.

22. Yang DS, Shewfelt RL, Lee K-S, Kays SJ: **Comparison of odor-active compounds from six distinctly different rice flavor types.** *Journal of Agricultural and Food Chemistry* 2008, **56:**2780-2787.
